# Supplementary material for: Polymer model integrates imaging and sequencing to reveal how nanoscale heterochromatin domains influence gene expression
Source: Nat Commun. 2025 Apr 23;16:3816. doi: 10.1038/s41467-025-59001-z (PMC12019571; doi:10.1038/s41467-025-59001-z)
Supplement: Supplementary file 1 — Supplementary Information [file 41467_2025_59001_MOESM1_ESM.pdf]

Supplementary information
for
Polymer Model Integrates Imaging and Sequencing to Reveal How Nanoscale Heterochromatin Domains Influence Gene Expression

Vinayak Vinayak[1,2], Ramin Basir[1,2], Rosela Golloshi[3,4], Joshua Toth[1,2], Lucas Sant'Anna[1,5], Melike Lakadamyali[1,6], Rachel Patton McCord[7], Vivek B Shenoy[1,2,*]

[1] Center for Engineering Mechanobiology, University of Pennsylvania, Philadelphia, PA 19104, USA
[2] Department of Materials Science and Engineering, University of Pennsylvania, Philadelphia, PA 19104, USA
[3] Departments of Cell Biology, Center for Cell Dynamics, Johns Hopkins University School of Medicine, Baltimore, MD 21218, USA
[4] Giovanis Institute for Translational Cell Biology, Johns Hopkins Medicine, Baltimore, MD 21218, USA
[5] Department of Bioengineering, Stanford University, Stanford, CA 94305, USA
[6] Department of Physiology, Perelman School of Medicine, University of Pennsylvania, Philadelphia, PA 19104, USA
[7] Department of Biochemistry & Cellular and Molecular Biology, University of Tennessee, Knoxville, TN 37996, USA
* Corresponding author; mail: vshenoy@seas.upenn.edu

Contents:

1. **Details of the polymer model**
    - **1.1. Building the chromatin polymer from sequencing data**
    - **1.2. Potentials used in the polymer model**
    - **1.3. Details of the executed dynamics**
    - **1.4. Detailed balance considerations**
    - **1.5. Running simulations and computing observable quantities**
    - **1.6. Analytical derivation of domain scaling**
    - **1.7. Motivating the epigenetic diffusion and epigenetic reactions**
2. **Cell-line specific simulations on data-informed polymers**
    - **2.1. Domain stability for other chromosomes of A375 and hMSC**
    - **2.2. Altered diffusion, acetylation and methylation runs**
    - **2.3. Memory analysis**
3. **STORM analysis**
    - **3.1. Imaging analysis workflow for calculating domain sizes**
    - **3.2. Data for domain size distribution of Y-27 treatment and tendinosis**
4. **Sequencing analysis**
    - **4.1. Analyzing the HiC map for A375 cells**
    - **4.2. RNA-seq analysis for A375 cells**

1 Details of the polymer model

1.1 Building the chromatin polymer

The polymer model presented in this study leverages experimental data to establish the initial configuration of the chromatin polymer. We utilize either ChIPseq in the form of Broad ChromHMM[2] tracks or experimentally obtained HiC data to determine the bead labels as euchromatin-like/active (blue) or heterochromatin-like/repressed (red). For the chromosomes of IMR90 and hMSC cell lines, we employ publicly accessible ChromHMM data for polymer initialization[3]. Specifically, we utilize the 15-state ChromHMM information and assign states 1-11 as blue beads corresponding to active states, while the remaining states are designated to red beads corresponding to repressed states. This strategy has been adapted from the CCM model[4]. Given that our bead size (10 kbp) is significantly larger than the 200 bp resolution of ChromHMM, we use a majority rule approach, where each bead is assigned to the state that occurs most frequently within its boundaries. Although this coarse-graining approach results in some loss of information, we observe that these regions predominantly belong to a single state, ensuring that the polymer faithfully represents the underlying epigenetic structure. For A375 cells, we utilize experimentally obtained HiC of the cells in control condition to construct the initial polymer. Here, we compute the first eigenvector (PC1) from the experimentally derived HiC contact map using cooltools[5] to delineate the A and B compartments. Beads falling within the A compartment are assigned as blue, while those falling within the B compartment are designated as red. More information on the Hi-C analysis is provided in section 4.1. All the polymer strands we form in the study are shown in Supplementary Fig. 1. Moreover, the sourcing and reason to use the different cells is depicted in Supplementary Table 1.

For our simulations, we chose a bead size (σ) of 65 nm corresponding to 10 kbp of contiguous chromatin region. A 10 kb region would dictate the number of nucleosomes of the order of ~50 contained in the bead. Using a lower limit, closed packing case, we can equate the volume of the bead to the volume of 50 nucleosomes, which would give us a lower bound of $\sigma =$ 40nm, assuming the size of nucleosomes to be ~10nm. For the upper bound, we can consider the polymer as a worm-like chain in which case we can get the size using the persistence and contour lengths as $\sigma^2 = 2L_C L_P - 2L_P^2$(assuming persistence length is significantly smaller than contour length, which is true for our case)[6]. Assuming a persistence length of ~1000 bp[7], we get $L_P = 6(16.5 + 10)$nm (with 16.5nm being the length of linker DNA and 10nm as the nucleosome size)[4]. This gives us an upper bound of ~500nm. Based on these estimates the choice we make here is in a physically reasonable range, if not a bit conservative. The conservative choice is reinforced by experimental observations made in Boettiger et. al.[8] where our bead size fits perfectly in scaling discovered. Moreover, another recent work[9] has shown that a 5kb region is well represented by a bead of size ~80 nm which falls in a similar range. To maintain a packing density consistent with experimentally observed ranges, the polymer is positioned within a fixed wall box, targeting a biologically relevant volume fraction of ~0.15[10, 11].

To understand the general physical principles driving the formation and growth of chromatin clutch domains, we also use a toy polymer model to elucidate the physical underpinnings of the proposed model. For this purpose, we choose a 3000-bead (~30Mbps) polymer and randomly

assign the bead labels to repressed (active) marks with a probability of 0.6 (0.4). This ratio has been chosen to account for the repressed majority (including the centromeric and telomeric regions) found in all the chromosomes we considered for the study.

In this work, we do not explicitly consider the role of loop formation through cohesin mediated loops. Even though they form a central part of chromatin organizational structure, we do not consider their role due to the following two reasons: a) Our current resolution is at 10kbps per bead and an average CTCF based cohesin loop is about ~50-100kbps. Hence it would be a loop only over 5-10 beads. b) CTCF ChIP-seq is not available for all the cell lines used and hence to keep our observations consistent, we did not use CTCF mediated loops for the cell lines we chose. We do not though, that our model does allow simple introduction of the loops in a static or dynamic manner. For the IMR90 cells, we ran simulations with static CTCF mediated loops which were added in a manner like Shi et. al. [4] and found that the obtained changes in epigenetic landscape did not show any differences from the case where such loops were not included. We do acknowledge that at a finer scale, the role of such loops will become more important as they can govern the local neighborhood of a chromatin segment, which ultimately determines the epigenetic fate of that segment.

1.2 Potentials used in the polymer model

We use the LAMMPS[12] engine to simulate our molecular dynamics system. The chromatin is modeled as a copolymer with euchromatin/A compartment/active regions represented as blue beads and heterochromatin/B compartment/repressed regions represented as red beads. The interaction between these beads is enforced using a Lennard-Jones potential as follows:

$$U_{LJ}^{\alpha\beta} = \begin{cases} 4\epsilon_{\alpha\beta}\left[\left(\frac{\sigma}{r}\right)^{12} - \left(\frac{\sigma}{r}\right)^{6}\right] & for \quad r \leq r_{cut} \\ 0 & for \quad r > r_{cut} \end{cases}$$

where α and β can be blue or red based on the ChIPseq/HiC annotation. The bead $\sigma = 1$ is chosen to be the same for both the beads. The radius for blue beads should be higher since the chromatin compaction is less but we keep it this way to make reduce model complexity. The interactions between red and blue beads are set such that the potential cutoff distance r_{cut} is set to $2^{1/6}\sigma$ resulting in excluded volume interactions also known as the WCA potential. The use of this potential is granted given the short-range interactions with respect to the length scale we have chosen for the system. The intra-cutoff r_{cut} among A or B beads is set at 1.8σ to account for bridging protein driven self-attraction. This leaves us with tuning the interaction energies between the heterochromatin-heterochromatin beads (ϵ_{HH}) and the euchromatin-euchromatin beads (ϵ_{EE}). Since heterochromatin is more compact that euchromatin, we add an additional constraint that $\epsilon_{EE} < \epsilon_{HH}$. In addition to this we have FENE (Finite Extensible Non-linear Elastic) potential mediated bonds in between neighboring beads on the linear polymer to maintain the integrity of the polymer:

$$U_{FENE}^{i} = -\frac{1}{2}K_{FENE}R_0^2 ln\left[1 - \left(\frac{|\boldsymbol{r}_{i+1} - \boldsymbol{r}_i|}{R_0}\right)^2\right]$$

where K_{FENE} is the spring constant and R_0 is the equilibrium bond length.

Combining the two, the net potential attributed to any bead i of the system can be described as the following function:

$$U_{net}^{i}(x) = \frac{1}{2}\sum_{j \neq i} U_{LJ}^{ij} + \sum_{j \in \{i-1, i+1\}} U_{FENE}^{j},$$

where i covers all the beads in the system. Since the persistence length of the chromatin fiber has been established to be about ~1000 bp[7] and our beads are much bigger than that, we ignore any polymer stiffness apart from the stiffness provided by excluded interactions between the beads.

To obtain the initial configuration of the polymer, we form a rod-like initial polymer using the process described in section SI1.1. This polymer is then arranged in a self-avoiding random walk. The Lennard-Jones potentials are turned on and the polymer is relaxed for 10^7 time steps to form the initial configuration. The potential energy plots, averaged over simulations show that the equilibration is reached (Supplementary Fig. 2). Since we have only two parameters to tune (ϵ_{HH} and ϵ_{EE}), with the constraint $\epsilon_{EE} < \epsilon_{HH}$, we use a hit-and-try methodology to analyze how well the obtained mean spatial distance vs. the genomic distance plots agree with the experimentally observed data[13] for IMR90 cells for chromosomes 20 and 21. Among the tested values we found $\epsilon_{EE} = 0.3$ and $\epsilon_{HH} = 1$ fit the experimentally observed values the best as shown in Supplementary Fig. 3. The polymer configuration is further validated through the Hi-C map presented in Fig 1e of the main text. To compute the Hi-C map, multiple polymer relaxations were executed, and any two beads were considered in contact if they were within 0.2 σ of each other. Thereafter the contact probability was calculated as a ratio of the number of simulations which maintained the contact. To match it with experimental observations[14], the experimental Hi-C data was normalized and changes to a log-scale.

It is important to note that the interaction parameters (ϵ_{HH} and ϵ_{EE}) are calibrated using experimental data to accurately reproduce the observed relationship between spatial and genomic distances. Although reducing A–A and B–B interaction strengths to lower values could preserve chromatin domain formation—even without active reactions—due to the interplay between polymer entropy and enthalpy, this would introduce inconsistencies between computational and experimentally observed chromatin organization length scales. We illustrate this effect in Supplementary Fig. 26 (corresponding to Fig. 1d), showing that lower interaction strengths fail to match experimentally observed length scales. In contrast, the potentials used in our model replicate these scaling relations, thus ensuring consistency with available experimental data.

1.3 Details on diffusion and reactions

We use Brownian Dynamics (shown in Supplementary Fig. 4a) to evolve the system in time in the presence of an implicit solvent, which in our case is nucleoplasm (water):

$$m\frac{d^2\boldsymbol{r}_i}{dt^2} = -\gamma\frac{d\boldsymbol{r}_i}{dt} - \nabla U_{net}^i(x) + \boldsymbol{\xi}_i$$

where γ is the friction coefficient and $\boldsymbol{\xi}_i$ is a stochastic noise obeying the fluctuation-dissipation relationship $\langle \xi_{i,\alpha}(t)\xi_{j,\beta}(t')\rangle = 2\gamma k_B T\delta_{i,j}\delta(t-t')\delta_{\alpha,\beta}$. Indices i,j run over particles and α,β run over the cartesian space. In this setting, using the Einstein relation, we obtain:

$$D = \frac{k_B T}{\gamma} = \frac{k_B T}{3\pi\eta\sigma},$$

where η is the viscosity of the background. Considering a bead size of 65 nm for 10 kbp region and a viscosity of $\eta = 150cP$ we can estimate corresponding Brownian time:

$$\tau_{Br} = \frac{\sigma^2}{D} = \frac{3\pi\eta\sigma^3}{k_B T} \approx 0.3\ s.$$

We utilize a velocity Verlet time integration methodology implemented within the LAMMPS engine in an to execute the trajectories in an NVE ensemble. Considering that we have chosen an integration time $\Delta t = 0.01\tau_{Br}$, we simulate approximately $\sim 1hr$ of real time corresponding to 1×10^6 integration time steps. This is the conversion have used for all the computations performed in this work.

Even though we use 150cp as our viscosity, the effective viscosity and the diffusion constant which the polymer encounters are different. To be more precise: In our simulations, each chromatin monomer is modeled as an individual molecule diffusing freely within the nucleoplasm. Consequently, we use the nucleoplasmic viscosity as the input parameter, rather than the higher apparent viscosity values associated with large-scale chromatin domains. Interactions between chromatin segments—mediated by epigenetic proteins—are explicitly included in our framework through chromatin-chromatin interaction terms. Furthermore, constrained monomer motion within the polymer is captured using finite extensible nonlinear elastic (FENE) potentials.

To quantify the effective viscosity and diffusivity in our model, we calculated the mean square displacement (MSD) of chromatin monomers and identified two distinct diffusion regimes (Supplementary Fig. 23)[15]. On shorter timescales (<10 seconds), we observed Brownian motion characterized by $\alpha \approx 1$ and $D_\alpha \approx 4 \times 10^{-3}\mu m^2/s$, corresponding to an effective viscosity of $\eta \approx 10^3 P$. On longer timescales (>10 seconds), the motion was sub-diffusive, with $\alpha \approx 0.5$ and $D_\alpha \approx 0.07\mu m^2/s$, yielding an effective viscosity of $\eta \approx 30P$. These values align well with the experimentally observed viscosity ranges for chromatin polymers and nuclear droplets, which span from ~1 P[16] to ~1000 P[17].

This parameterization ensures that our simulations accurately capture chromatin's dynamic behavior, balancing computational feasibility with biological realism. By emphasizing the interaction-driven dynamics rather than relying solely on viscosity adjustments, our approach provides a mechanistic basis for chromatin mobility while remaining consistent with observed experimental values.

Next, the epigenetic diffusion is modeled like Kawasaki dynamics studied in Ising spin systems (Supplementary Fig. 4b)[18]. We choose an epigenetically conservative exchange

process since diffusion does not alter the ratio of hetero-to-euchromatin but drives the system to a lower energy state through epigenetic redistribution. Each exchange between the epigenetic marks (colors) of the beads is modeled using a Metropolis criterion which is dictated by the acceptance probability of exchange as:

$$p(s \to s') = \min(1, e^{-\Delta E/k_B T})$$

where ΔE is the energy difference between the final (after epigenetic exchange) and the initial (before epigenetic exchange) configuration. The temperature T is the same as the Brownian simulation to maintain detailed balance and not introduce any out-of-equilibrium phenomenon. This is explained in detail in the following section.

The epigenetic reactions account for microenvironment-driven methylation (Γ_{me}) and acetylation (Γ_{ac}) of the chromatin. They are modeled as a Monte-Carlo processes where any bead can be randomly chosen, and its epigenetic marking can be changed from euchromatin to heterochromatin with a probability equal to Γ_{ac}/Γ_{me} and vice versa (Supplementary Fig. 4c). This introduces non-equilibrium phenomenon by breaking detailed balance as discussed in the following section.

1.4 Accounting for detailed balance in the simulations

Chromatin dynamics in our model are driven by passive diffusion and active epigenetic reactions. It is central to our finding that the out-of-equilibrium epigenetic remodeling is driven solely by epigenetic reactions. To show our proposed dynamics follow these constrains, here we show how well our model follows equilibrium criteria using detailed balance. We use the Kolmogorov criteria to test detailed balance conditions. According to the criteria, for a system exhibiting detailed balance, the transition rates over any closed loop of states of the system should be independent of the direction in which the loop is traversed. We first account for our choice of the temperature of the Brownian dynamics to be the same as the epigenetic diffusion. As shown in Supplementary Fig. 5(left), we consider a system of four particles which are acted upon by Lennard-Jones interactions as described in the previous sections. The initial and final state are the same, thereby constituting a loop. We only consider the effect of diffusion of epigenetic marks and Brownian motion and quantify the probability of each step using $\exp(-\Delta E/kT_x)$, where ΔE is the energy required for the process and T_x is the temperature for the Brownian movement or epigenetic diffusion. T_x can be T_E for the case when we are attempting an epigenetic diffusion move and T_{Br} for the case of Brownian diffusion. As can be seen, the clockwise (forward) loop in Supplementary Fig. 5 (left panel, it has the following rate:

$$P_{clock} = \exp\left(-\frac{2\epsilon}{k_B T_E}\right)$$

Contrary to this, the rate of the anti-clockwise (backward) loop is given by:

$$P_{anti-clock} = \exp\left(-\frac{\epsilon}{k_B T_E}\right)\exp\left(-\frac{\epsilon}{k_B T_{Br}}\right)$$

where T_{Br} is the Brownian temperature. Detailed balance holds true only if $P_{clock} = P_{anti-clock}$. Hence for detailed balance to hold, T_E must be the same as T_{Br}. A simpler phenomenological

argument to understand why this must be true is as follows: In a canonical ensemble picture, the Brownian dynamics are driven by a heat bath at temperature T_{Br} and the epigenetic exchange by a bath at temperature T_E. If the two temperatures are not equal, then a constant energy flux is established between the two baths, making it an active system.

Using a similar scheme, we next argue how epigenetic reactions break detailed balance in our model setup. We consider a system with two particles which are initially epigenetically distinct and proceed as shown in Supplementary Fig. 5 (right panel). As is clear from the figure the clockwise rate is given by $P_{clock} = \Gamma_{me}\Gamma_{ac}\exp\left(-\frac{\epsilon}{k_B T_{Br}}\right)$ while the anti-clockwise loop rate is equal to $P_{anti-clock} = \Gamma_{me}\Gamma_{ac}$. Hence, irrespective of the value of the reactions, they will always break detailed balance in the current setup as $P_{clock} \neq P_{anti-clock}$. Hence it is only the reactions which introduce an out-of-equilibrium aspect to our model while the diffusions maintain detailed balance.

1.5 Running simulations and computing observable quantities

With the initial configuration in SI1.2, we decide on the reaction rates as described in the main text and run simulations generally for a 2 hour time point or 10 hour time point, corresponding to 2×10^6 integration steps or 10×10^6 integration steps respectively. Simulations are repeated over different initial configurations to establish statistical significance and to capture the effects of cellular heterogeneity. Our analysis consists of both, analysis over single simulations and over multiple simulations based on the context. Broadly we use the following analysis strategies to observe key observables in our systems:

1. Measuring the radius of gyration of the heterochromatin-rich packing domains (Supplementary Fig. 6): To identify the domains, we first isolate the heterochromatin (red) beads from our polymer. Thereafter, we run the DBscan clustering algorithm, available through the scikit-learn package[19] in python. For the implementation, we use an eps=1.2 and the minimum number of particles in a cluster as 10. Then we compute the radius of gyration of the obtained domains. We note here that for all the cases where we plot the distribution of their domains over all obtain clusters except for the case with the prototype polymer.
2. Producing kymographs and distance maps to analyze the spatiotemporal evolution of epigenetic marks: We use simple matplotlib based scatter plots to dhow both the kymographs and distance plots. To produce the kymographs, we do not average over multiple simulations as the aim is to show single cell scale epigenetic evolution while for distance maps multiple simulations are averaged over.
3. Producing polymer figures: We use VMD[20] to visualize the polymer 3D configurations.

1.6 Analytical derivation of domain scaling

Here we take a nuanced approach wherein we try to show the scaling relation through a simple dimensional analysis argument. As shown in Supplementary Fig. 7, across the boundary of a heterochromatin rich domain, there are two fluxes which are balancing the size of the domain:

1. The diffusion of the heterochromatin into the domain: The flow of heterochromatin into the domain can be quantified as the inward flux times the surface area of the sphere. To calculate the flux, we turn to Fick's law, which dictates that:
$$J = -D\frac{d\phi}{dr} = -D\frac{[concentration]}{[length\ scale]}$$
Where J is the flux, D is the diffusion coefficient and $d\phi/dr$ is the heterochromatin concentration gradient. More precisely, we need to realize the best estimates for the concertation and relevant length scale in the system to arrive at a reasonable empirical relation for the flux. Let us assume that due to the formation of a depletion layer right outside the heterochromatin domain, the heterochromatin content is nil. Therefore, the flux now only depends on the far-field concentration. In this case, the far field concentration will be the average heterochromatin content in the system which is $\frac{\Gamma_{me}}{\Gamma_{me}+\Gamma_{ac}}$. The only relevant length scale in the diffusion problem is the radius of the sphere and hence we can estimate the flux as (ignoring the sign):
$$J = D\frac{1}{R}\frac{\Gamma_{me}}{\Gamma_{me}+\Gamma_{ac}}$$
This will dictate that the net inflow of heterochromatin can be estimated as:
$$Influx\ of\ heterochromatin = 4\pi R^2 \times J = 4\pi RD\frac{\Gamma_{me}}{\Gamma_{me}+\Gamma_{ac}}$$
2. The conversion of heterochromatin to euchromatin within the domain due to acetylation reaction: We firstly assume a uniform concentration of heterochromatin within the formed domain, C. Thereafter, since the reactions occur uniformly over the whole space in our setting, we can estimate the outflux of heterochromatin as:
$$Outflux\ of\ heterochromaitn = Vol.of\ sphere \times rate\ of\ acetylation \times C = \frac{4}{3}\pi R^3\Gamma_{ac}C$$

Equating the outflux with the influx we obtain:
$$R^2 \propto \frac{\mathrm{D}\,\Gamma_{me}}{\Gamma_{ac}(\Gamma_{me}+\Gamma_{ac})}$$
The extended derivation for the domain scaling is provided in the SI of Heo et. al.[21] or Kant et. al.[22].

1.7 Motivating epigenetic diffusion and reactions

Epigenetic marks, in the context of our current model, are established through enzymatic reactions leading to changes in acetyl and methyl group deposition on lysine residues of histones. While these processes are fundamentally active, they also exhibit a neighborhood-dependent nature, where the local chromatin environment significantly influences the activity of histone-modifying enzymes. Here we explain the basis for the neighborhood dependent action of these enzymes and elucidate how the interplay between enzymatic activity and neighboring chromatin state underpins the basis for incorporating diffusion-like behavior into the modeling of epigenetic dynamics.

Biological Context for Neighborhood-Dependent Dynamics: The activity of histone-modifying enzymes, such as histone methyltransferases (HMTs), acetyltransferases (HATs), deacetylases (HDACs), and demethylases (HDMs), depends on two factors:

1. Epigenetic Makeup of the Neighborhood: The energy required for enzymatic interconversion of marks (e.g., switching from a methylated to acetylated state or vice versa) is dictated by the surrounding chromatin state. For instance, converting a methylated locus to an acetylated one in a methylation-rich environment necessitates overcoming stronger stabilizing interactions (e.g., HP1-mediated or other chromatin interactions) compared to a similar conversion in an acetylation-rich environment (Supplementary Fig. 25b). This introduces an energy barrier that determines the likelihood of epigenetic state transitions.

   Mathematically, this dependency can be expressed using a probability-based framework:

$$P_{R \to B} \propto e^{-\beta \Delta E_{R \to B}}$$
$$P_{B \to R} \propto e^{-\beta \Delta E_{B \to R}}$$

   where $\Delta E_{R \to B}$ and $\Delta E_{B \to R}$, are the energy differences for red-to-blue and blue-to-red conversions, respectively, and β represents the inverse temperature. This framework ensures that enzymatic transitions are energetically driven and neighborhood-dependent, leading to coarsening of epigenetic marks into domains of similar states(Supplementary Fig. 25b).

2. Intrinsic Activity of Remodeler Enzymes: The rate of epigenetic changes is also influenced by the nuclear concentration and enzymatic efficiency of remodelers. For example, increased concentrations of HMTs lead to accelerated methylation, while different remodelers exhibit varying activity levels due to inherent differences in enzymatic properties. These variations can be incorporated into the model by introducing an additional multiplicative term α that modifies the transition probabilities:

$$P_{B \to R} \propto \alpha e^{-\beta \Delta E_{B \to R}}$$

   Here, α captures the influence of factors such as nuclear enzyme concentration, microenvironmental changes, or drug-induced modifications.

Rationale for Incorporating Epigenetic Diffusion and reactions:
To capture the combined effects of neighborhood-dependent enzymatic activity and intrinsic enzymatic rates, a kinetic Monte Carlo (KMC) framework is employed (Supplementary Fig 25a). Simulations using this framework reveal the following key insights:

1. **Steady-State Domain Formation**: Finite-sized epigenetic domains emerge at equilibrium, consistent with experimentally observed chromatin organization (Supplementary Fig. 25c).
2. **Intermediate Dynamics**: During transient stages, the system exhibits effective conservative exchange of epigenetic marks, analogous to Kawasaki dynamics, where mark interconversion occurs without net loss or gain (Supplementary Fig. 25d,e).

This approach enables the decomposition of epigenetic dynamics into two distinct components:

- A **conservative, neighborhood-dependent diffusion term**, reflecting the energetically driven exchange of epigenetic marks.

- A **non-conservative reaction term**, representing uniform, active processes such as enzymatic deposition of marks.

This decomposition into conservative and non-conservative components provides two major advantages:

- It distinguishes between energetically driven and active processes, allowing for more accurate modeling of chromatin dynamics.
- It accommodates uniform changes across the system, enabling simulations of treatment-induced alterations without positional bias.

Summary and Implications:

The inclusion of diffusion-like behavior for epigenetic marks is rooted in the biological reality of neighborhood-dependent enzymatic activity. By leveraging a KMC framework and incorporating a local energy landscape we show that the model we propose captures the emergent dynamics of chromatin organization, offering a biologically informed perspective on epigenetic remodeling. Our diffusion-reaction driven approach not only aligns with observed phenomena but also provides a versatile platform for simulating diverse perturbations, such as drug treatments or enzyme mutations, within the chromatin context by tweaking the epigenetic reactions alone.

2. Cell-line specific simulations on data-informed polymers

2.1 Domain stability for other chromosomes of A375 and hMSC

In the scenario where the nuclear concentration of epigenetic/histone remodelers is constant, it is imperative to show that our model can maintain the 3d chromatin organization and epigenetic distribution, which we quantify through the kymograph (representative of the epigenomic stability) and the distance matrix (representative of the spatial stability). In the main text, we showed the kymograph of chromosome 19 of A375 cells for ~2 hours of real time. Here we extend that observation to account for the chromatin accessibility. We show the distance matrix for chromosomes 18-21 of A375 cells for about ~2 hours of real time, over which we have done majority of analysis for our paper in Supplementary Fig. 8. Thereafter, we show the epigenetic stability of these chromosomes for ~10 hours of real time in Supplementary Fig. 9.

Lastly to ensure that such a trend is observed across cell lines, we repeat this analysis for hMSC cells with the distance matrices Supplementary Fig. 10 and kymographs in Supplementary Fig. 11. This analysis ensures that in the case where there are no shifts in the average nuclear epigenomic makeup, the chromatin will sustain its epigenomic and pair-wise spatial identity. Since the epigenomics remain stable through the time, this will also ascertain that the transcriptional activity of the cell also remains stable in the interphase time window.

2.2 Altered Diffusion, Acetylation and Methylation runs

One of the key parameters for our simulations is the ratio of the diffusion to reaction rate, i.e. D/Γ_{ac}. We have already quantified the evolution of the 3D chromatin organization with respect to this parameter in the main text and explained its origin in SI1.6. Here we analyze the evolution of the epigenetic marks on the chromatin strand of A375 cells as a function of his parameter in Supplementary Fig. 12. The observations are like observations of the mesoscale observations

wherein the epigenetic distribution randomizes completely as the reaction rates increase while we observe almost complete Ostwald ripening as the diffusion rates increase.

With a change in the average nuclear concentration of the epigenetic regulators, the epigenetic makeup of the chromatin strand changes. We already show the chr19 specific changes after acetylation for A375 in the main text. Here we extend those results to show how the changes are replicated in other chromosomes of A375 and hMSCs which are central to our study. For A375, we firstly show how the epigenetic evolution of the epigenetic marks on acetylation and methylation through kymographs in Supplementary Fig. 13. These figures suggest that major chromatin epigenetic changes are constrained to the domain boundaries. To cement this observation, we plot the distance matrices for acetylation and methylation in Supplementary Fig. 14. We also plot the difference matrices for chromosome 19 in Supplementary Fig. 27. To drive home the concept of localization of epigenetic changes to the domain boundaries we further analyze the methylation of chromosome 19 and acetylation and methylation of chromosome 20 in more detail. Thus this analysis confirms that chromatin domains change epigenetically.

2.3 Memory analysis

For the memory analysis, there are two primary parameters which we could vary: i) The total time and ii) The magnitude of the reaction rates. In our would we focus on the later and keep the total time a constant. We do this mainly since we are bound by the timescale of the interphase of a cell which is ~10 hours and our attempt is to see how the organization changes in that timescale. We perform this analysis for chromosome 20 of hMSC cells. The plots for the varying epigenetic reaction rates are plotted in Supplementary Fig. 15.

Our model can be combined with chromatin replication models and longer timescale observations could be made as well but it lies beyond the scope of the current model.

3. STORM analysis

3.1 Computing domains from STORM images

The images obtained from ONI STORM are first processed using the Nanoimager (ONI) software to perform drift correctio. Thereafter, the images are imported to a custom written code on MATLAB code which we have used in our previous publication. Voronoi-tessellation is used for density quantification and construction of a Voronoi polygon of each localization. Then the density corresponding to each polygon is approximated using the inverse area of the polygons. Based on custom threshold, the nucleus is divided into heterochromatin and euchromatin rich regions and the heterochromatin rich areas are conserved. This threshold value is determined such that > 60 percentile of the density distribution fall into the heterochromatin regime and the threshold is conserved through the different replicates. It is important to note that the density threshold is kept same for a treated sample and its corresponding control. Density-Based Spatial Clustering of Applications with Noise (DBSCAN) is then used to obtain the heterochromatin domains from the underlying point cloud distribution. The radii of these domains are what we refer to as the domains in our imaging analysis.

We illustrate this workflow in Supplementary Fig. 16. We plot one replicate of each of the control and TSA treated cells and along with that plot the DBSCAN detected interior domains

(shown in black) for each of the cell. Our workflow can also detect lamina associated domains, which are excluded from our analysis in this work[1]. The domain size distributions for the interior domains show the Control-vs-TSA trends which have been presented in the main text. Similar analysis has been repeated for cells on stiff and soft gels and GSK treatment.

3.2 Data for domain size distribution of Y-27 treatment and tendinosis

The data for Y-27 and tendinosis is obtained from Heo et. al. [21] while the analysis is followed from DGK et. al. [1] and plotted in Supplementary Fig. 17.

4. Sequencing analysis

4.1 Analyzing the HiC map and corresponding simulations for A375 cells

To obtain the A/B compartments, the two replicates for the control and TSA treated cells were combined to get a more robust Hi-C signal. Thereafter principle component analysis was performed using cooltools[23] was used to delineate the A and B compartments and to obtain the PC1 scores on 10kb binned matrices, 40kb binned matrices. The 10kb compartmentalization was used to initialize the chromatin polymer and the 40kb was used for compartment switching analysis. The Hi-C contact maps hardly show any differences between the control and TSA treated cells as shown in Supplementary Fig. 18, 19. In Supplementary Fig. 20, we plot the compartment changes over other chromosomes chr18-21 to show that the compartment changes are a boundary phenomenon.

<u>Calculating the flipping score from the simulations and matching with simulations:</u> The flipping score was calculated by averaging scores across simulations. Initially, each chromatin segment is assigned a score of 0. For each simulation, segments changing to blue are assigned +1, while those changing to red are assigned -1. The scores are then summed across multiple simulations and averaged. The final flipping score represents the frequency of mark changes for each segment. An absolute threshold of 0.5 is applied to determine significance, indicating a segment has changed its mark in at least half of the simulations. Finally, the scores are compared to experimental results to assess their correlation.

4.2 RNA-seq analysis for A375 cells

The RNA-seq data for the three replicates was combined and analyzed using DESeq2 followed by GO enrichment analysis from cluster profiler to get the differentially regulated pathways. The differential pathway analysis was repeated in the following two settings:

1. For all the genes (plotted in the main text)
2. For genes within 300kb of the domain boundaries (plotted in Supplementary Fig. 21)

Additionally, we have also plotted the differentially expressed genes on chr19 in Supplementary Fig. 22 which shows that differentially regulated genes are close to domain boundaries.

Lastly, we found that Wnt signaling pathway (GO:0060070) and positive regulation of stress-activated MAPK cascade (GO:0051403) were both enriched within 300kb more than

outside of it. The gene lists from Supplementary Table 2 provide the support for the percentages we plot in the main text.

References:

1. Dhankhar, M., et al., *Revealing the Biophysics of Lamina-Associated Domain Formation by Integrating Theoretical Modeling and High-Resolution Imaging.* bioRxiv, 2024.
2. Ernst, J. and M. Kellis, *ChromHMM: automating chromatin-state discovery and characterization.* Nat Methods, 2012. **9**(3): p. 215-6.
3. Bernstein, B.E., et al., *The NIH Roadmap Epigenomics Mapping Consortium.* Nat Biotechnol, 2010. **28**(10): p. 1045-8.
4. Shi, G., et al., *Interphase human chromosome exhibits out of equilibrium glassy dynamics.* Nat Commun, 2018. **9**(1): p. 3161.
5. Open2C, et al., *Cooltools: enabling high-resolution Hi-C analysis in Python.* BioRxiv, 2022: p. 2022.10. 31.514564.
6. Kroon-Batenburg, L.M.J., et al., *Estimation of the Persistence Length of Polymers by MD Simulations on Small Fragments in Solution. Application to Cellulose.* The Journal of Physical Chemistry B, 1997. **101**(42): p. 8454-8459.
7. Sanborn, A.L., et al., *Chromatin extrusion explains key features of loop and domain formation in wild-type and engineered genomes.* Proc Natl Acad Sci U S A, 2015. **112**(47): p. E6456-65.
8. Boettiger, A.N., et al., *Super-resolution imaging reveals distinct chromatin folding for different epigenetic states.* Nature, 2016. **529**(7586): p. 418-22.
9. Kadam, S., et al., *Predicting scale-dependent chromatin polymer properties from systematic coarse-graining.* Nat Commun, 2023. **14**(1): p. 4108.
10. Paulsen, J., et al., *Chrom3D: three-dimensional genome modeling from Hi-C and nuclear lamin-genome contacts.* Genome Biol, 2017. **18**(1): p. 21.
11. Dekker, J. and T. Misteli, *Long-Range Chromatin Interactions.* Cold Spring Harb Perspect Biol, 2015. **7**(10): p. a019356.
12. Thompson, A.P., et al., *LAMMPS - a flexible simulation tool for particle-based materials modeling at the atomic, meso, and continuum scales.* Computer Physics Communications, 2022. **271**: p. 108171.
13. Wang, S., et al., *Spatial organization of chromatin domains and compartments in single chromosomes.* Science, 2016. **353**(6299): p. 598-602.
14. Rao, S.S., et al., *A 3D map of the human genome at kilobase resolution reveals principles of chromatin looping.* Cell, 2014. **159**(7): p. 1665-80.
15. Brahmachari, S., et al., *Temporally Correlated Active Forces Drive Segregation and Enhanced Dynamics in Chromosome Polymers.* PRX Life, 2024. **2**(3): p. 033003.
16. Nicklas, R.B., *Measurements of the force produced by the mitotic spindle in anaphase.* J Cell Biol, 1983. **97**(2): p. 542-8.
17. Caragine, C.M., S.C. Haley, and A. Zidovska, *Surface Fluctuations and Coalescence of Nucleolar Droplets in the Human Cell Nucleus.* Phys Rev Lett, 2018. **121**(14): p. 148101.
18. Resende, D.C., F.C. Cacüador, and S.O. Dantas, *Kawasaki Dynamics on Bidimensional Spin Systems.* Physics Procedia, 2012. **28**: p. 105-111.
19. Pedregosa, F., et al., *Scikit-learn: Machine learning in Python.* the Journal of machine Learning research, 2011. **12**: p. 2825-2830.
20. Humphrey, W., A. Dalke, and K. Schulten, *VMD: visual molecular dynamics.* J Mol Graph, 1996. **14**(1): p. 33-8, 27-8.

21. Heo, S.J., et al., *Aberrant chromatin reorganization in cells from diseased fibrous connective tissue in response to altered chemomechanical cues.* Nat Biomed Eng, 2023. **7**(2): p. 177-191.
22. Kant, A., et al., *Active transcription and epigenetic reactions synergistically regulate meso-scale genomic organization.* Nat Commun, 2024. **15**(1): p. 4338.
23. Open2C, et al., *Cooltools: Enabling high-resolution Hi-C analysis in Python.* PLoS Comput Biol, 2024. **20**(5): p. e1012067.

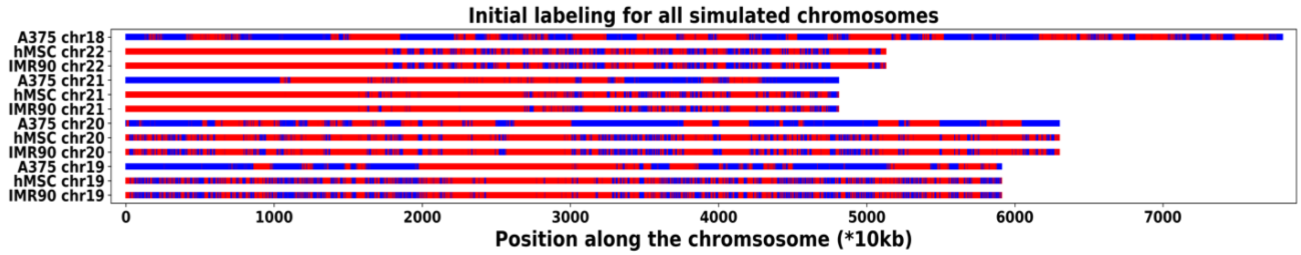

Figure 1: Initial labeling of all simulated chromosomes.

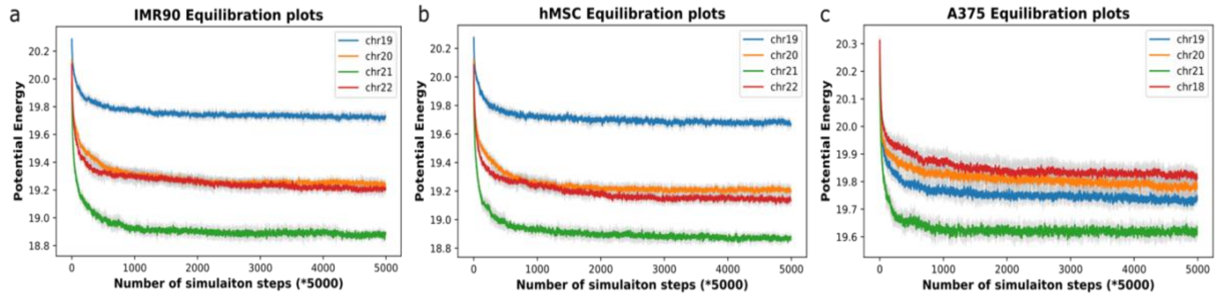

Figure 2: Relaxation plots for all the simulated chromosomes. The plots show *mean* +/- SD across 10 simulations for all simulated chromosomes for (a) IMR90, (b) hMSC and (c) A375 cells.

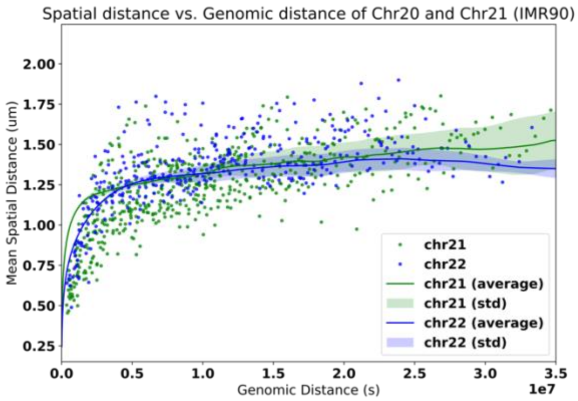

Figure 3: Experimental validation of the chosen potentials. The scatter represents the experimentally observed values[1] while the lines and the shaded regions represents the simulated fit. The plots show *mean* +/- SD across 10 simulations.

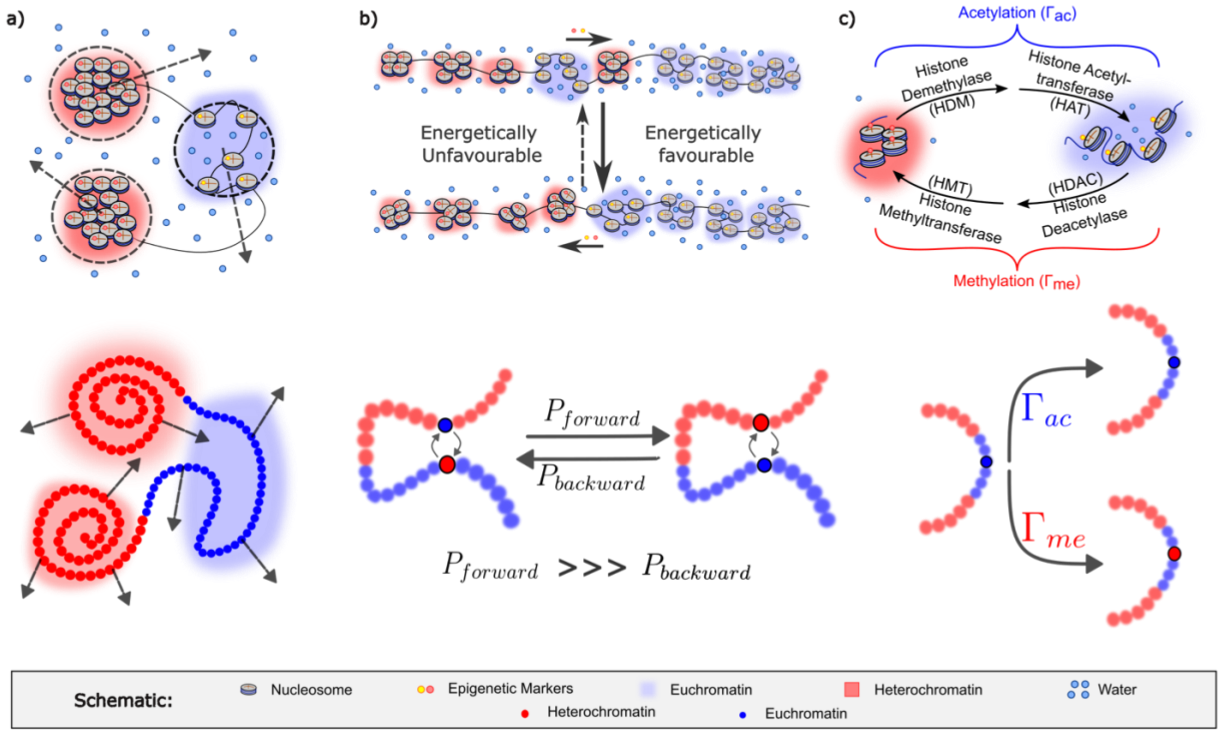

Figure 4: Executed dynamics and corresponding segment-scale polymer representations of the dynamics. a) Represents Brownian motion, b) represents diffusion of epigenetic marks and c) shows execution of epigenetic reactions

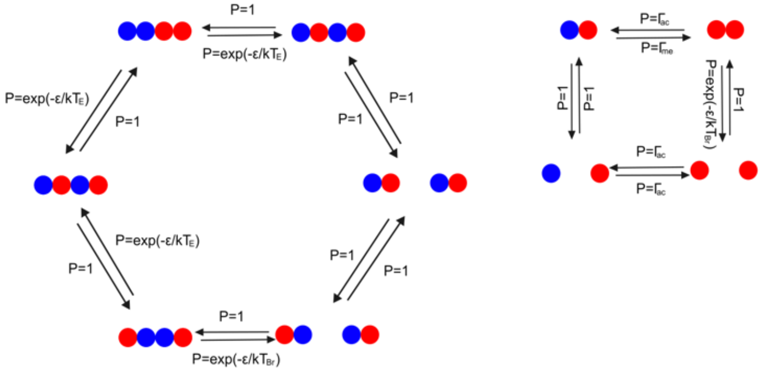

Figure 5: Diffusion processes maintain detailed balance and reactions introduce out-of-equilibrium phenomenon. The left panel shows detailed balance is followed for diffusion and the clockwise and the anti-clockwise probabilities are the same. The right panel shows that detailed balance is broken with the introduction of reactions.

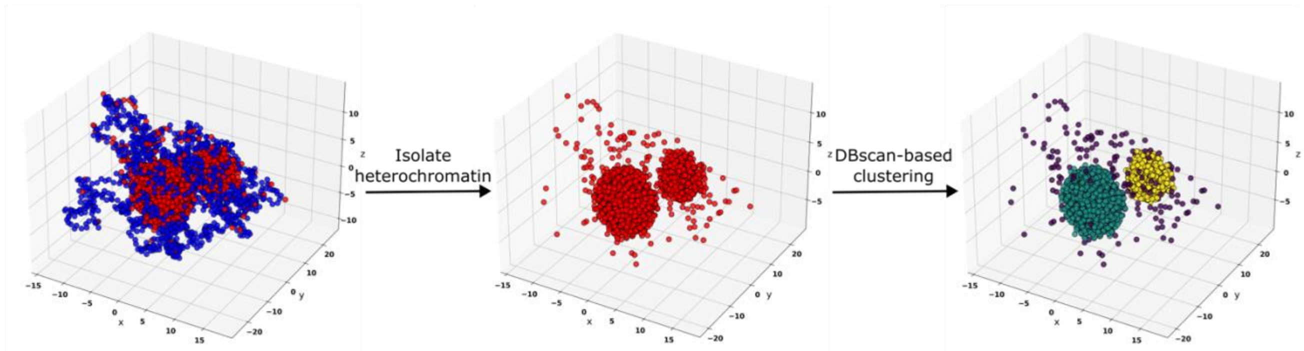

Figure 6: Computing the radius of gyration of the domains involves isolating the heterochromatin regions followed by clustering using DBSCAN.

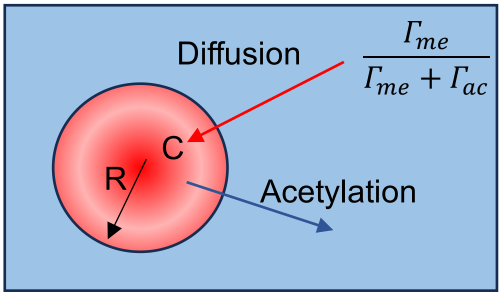

Figure 7: Representative diffusion-reaction set-up at steady state

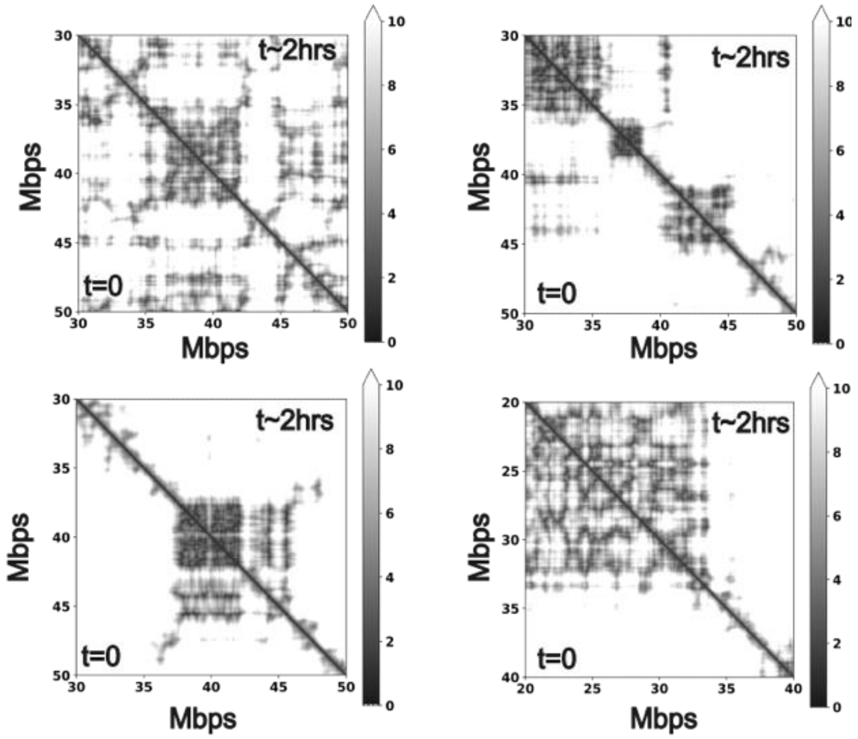

Figure 8: A375 chromosomes show stable point-to-point distances in 2 hours of real time. Chr18, 19, 21 and 20 are placed clockwise. The scale is 100nm. Plots were obtained by averaging across 10 simulations.

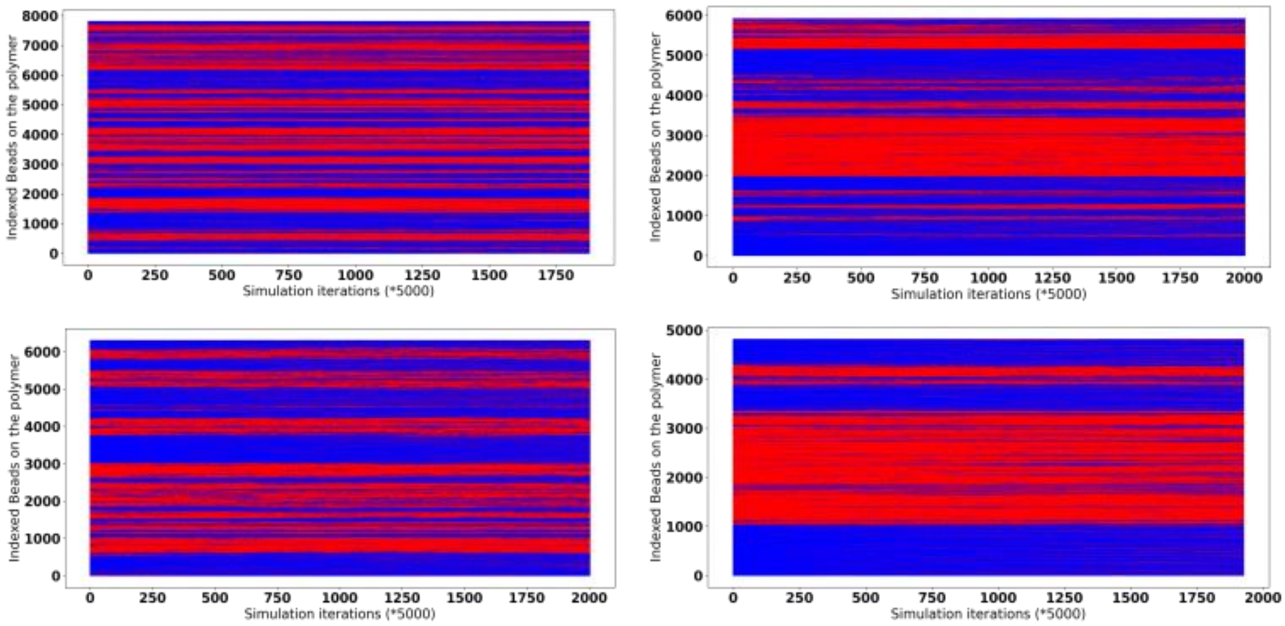

Figure 9: Domain stability over 10 hours of simulation for A375 chromosomes. Chr 18, 19, 21 and 22 are placed clockwise.

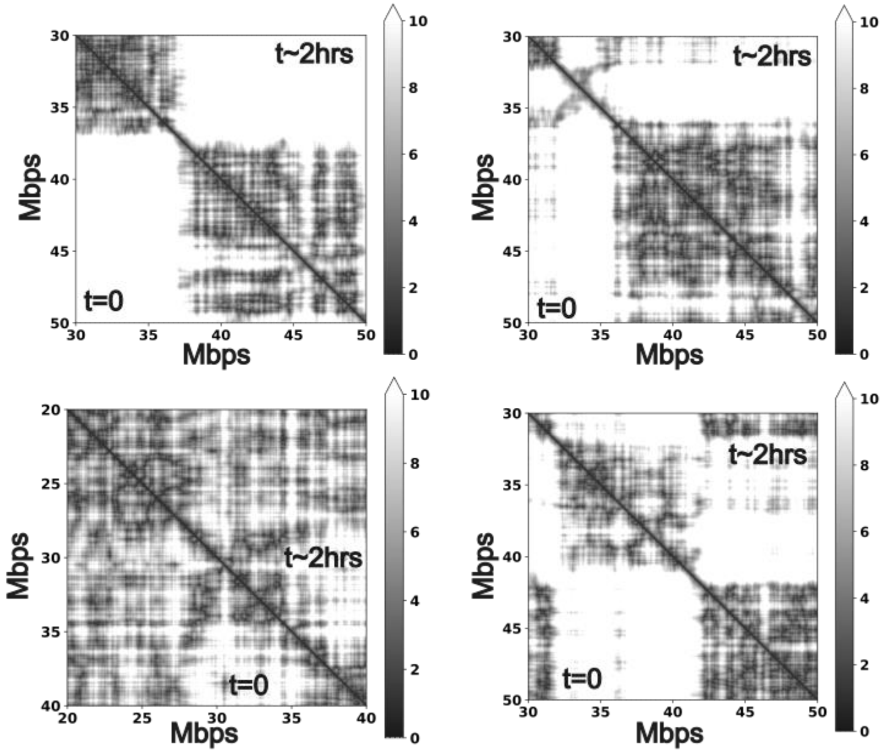

Figure 10: hMSC chromosomes show stable point-to-point distances in 2 hours of real time. Chr19, 20, 22 and 21 are placed clockwise. The scale is 100nm. Plots were obtained by averaging across 10 simulations.

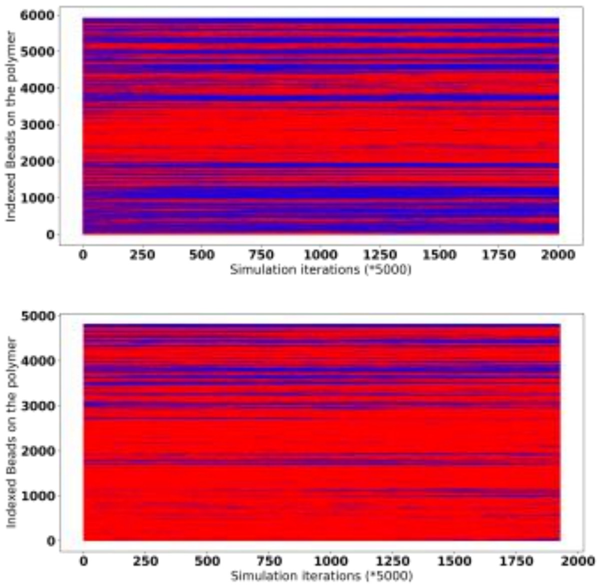

Figure 11: Domain stability over 10 hours of simulation for hMSC chromosomes. Chr 19, 20, 22 and 21 are places clockwise.

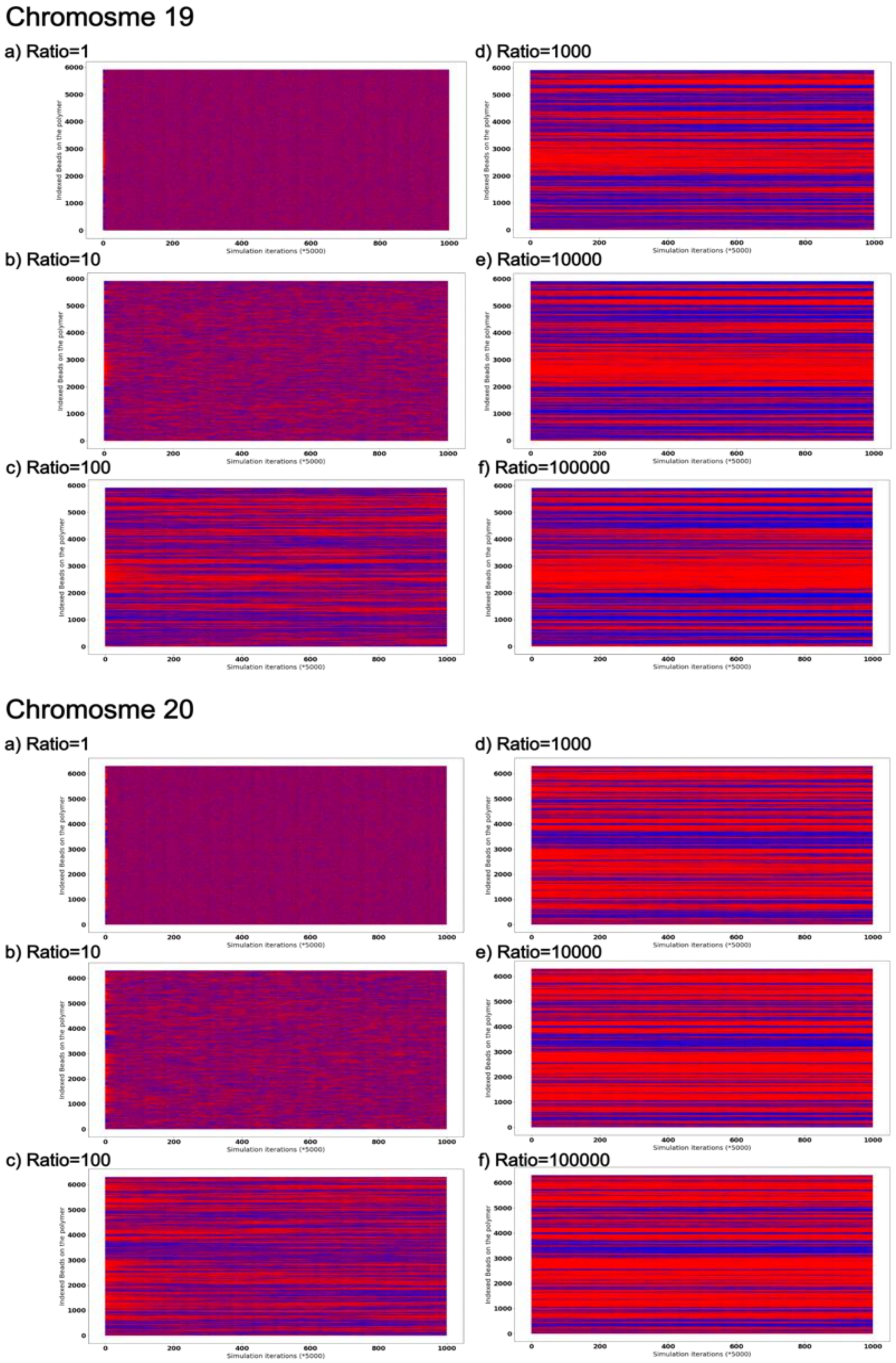

Figure 12: Kymographs of evolution of the chromatin domains on the linear polymer for chromosomes 19 and 20 of hMSC. The plots show the evolution of the domains with time at different ratios of number of steps at which epigenetic reaction takes place to the number of steps at which epigenetic diffusion takes place.

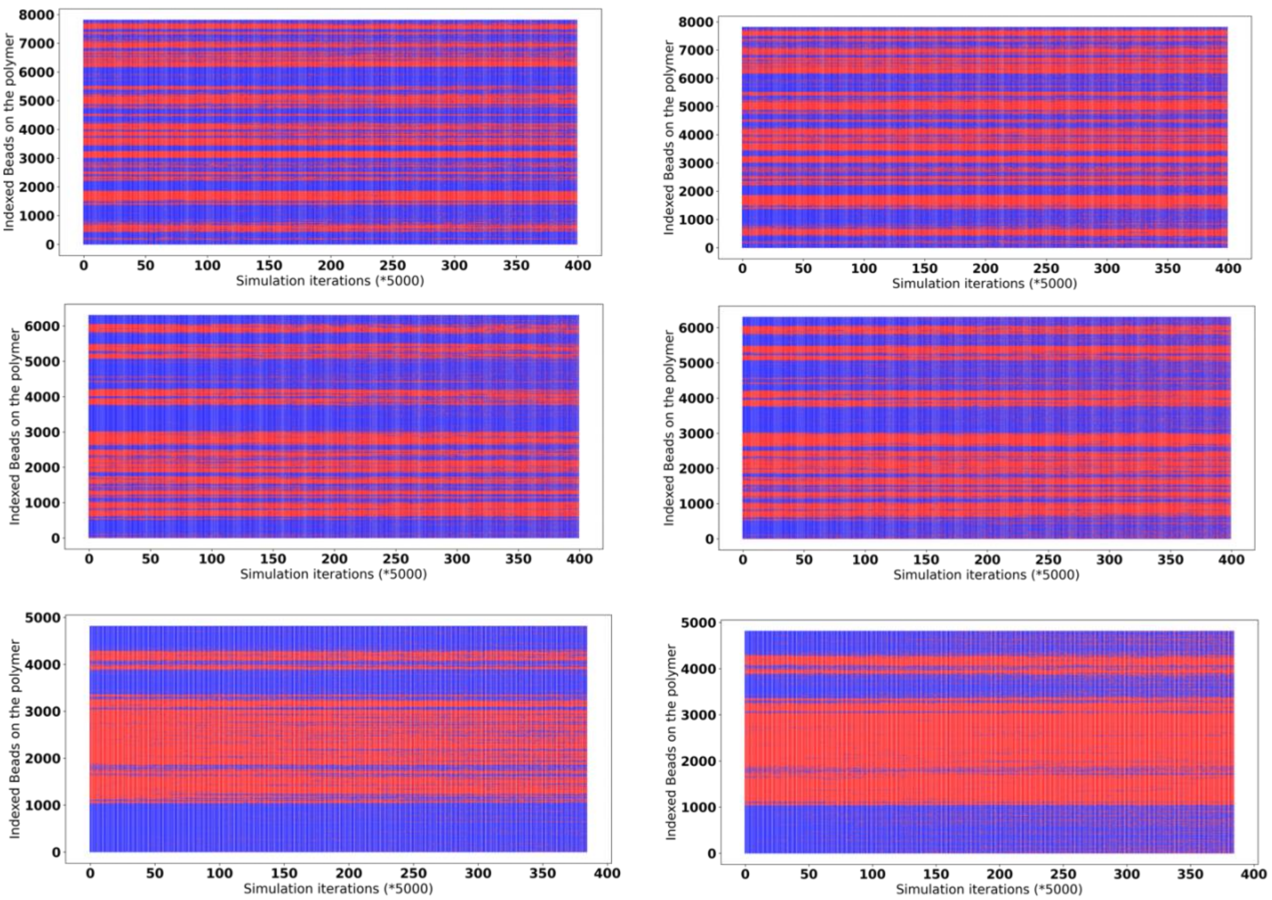

Figure 13: Kymographs of evolution of the epigenetic landscape for A375 chromosomes. Each row is a single chromosome with higher acetylation (Γ_{me}/Γ_{ac} =0.2) in the left panel and higher methylation (Γ_{me}/Γ_{ac} =0.9) in the right panel. From top to bottom: Chr18, Chr20 and Chr22.

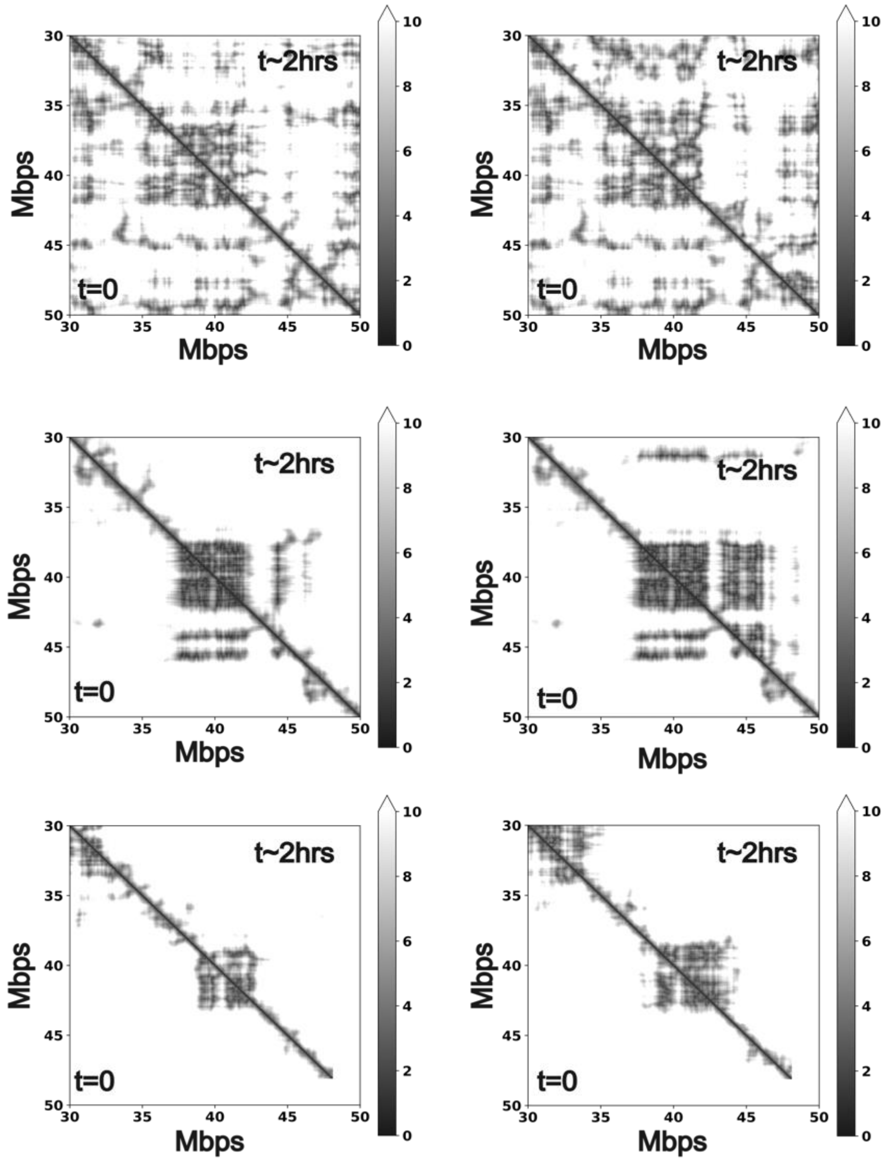

Figure 14: A375 distance maps for acetylation and methylation. Each row is a single chromosome with the left panel showing acetylation (Γ_{me}/Γ_{ac} =0.2) and the right panel showing methylation(Γ_{me}/Γ_{ac} =0.9). From top to bottom: Chr18, Chr20, Chr21. The scale is 100nm. Plots were obtained by averaging across 10 simulations.

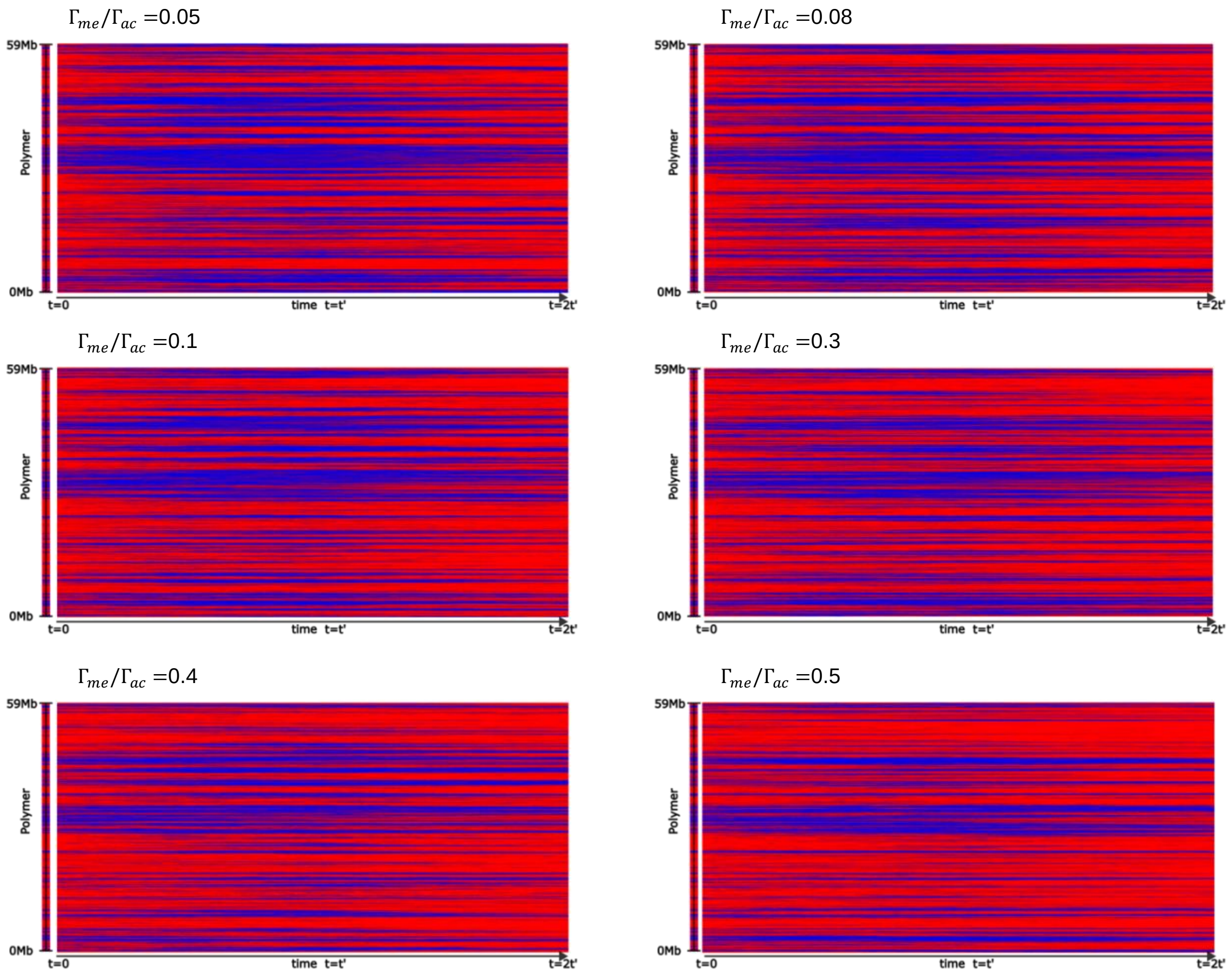

Figure 15: Memory runs for hMSC Chr20. For all runs the initial and final reaction ratios are the same to represent the initial conditions (Γ_{me}/Γ_{ac}). At the intermediate point the ratio is (Γ_{me}/Γ_{ac}) is mentioned with the plot.

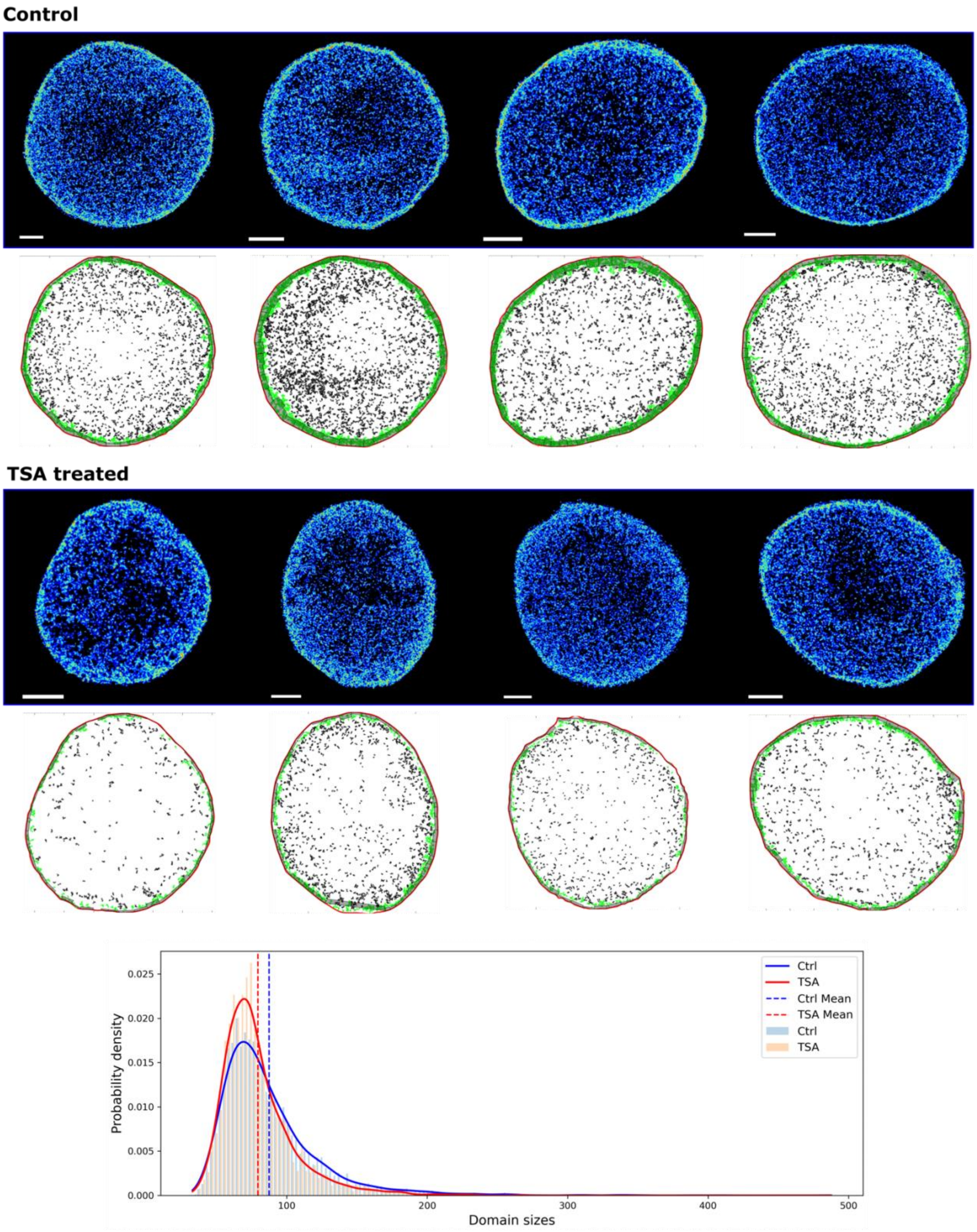

Figure 16: STORM analysis for control and TSA treated A375 cells. For each cell, shown in the black panel, its corresponding DBSCAN detected interior domains are shown as black clusters. The lamin associated domains (in green boxes) are excluded from the analysis. The final size distributions for the shown images are provided in the bottom panel along with the means.

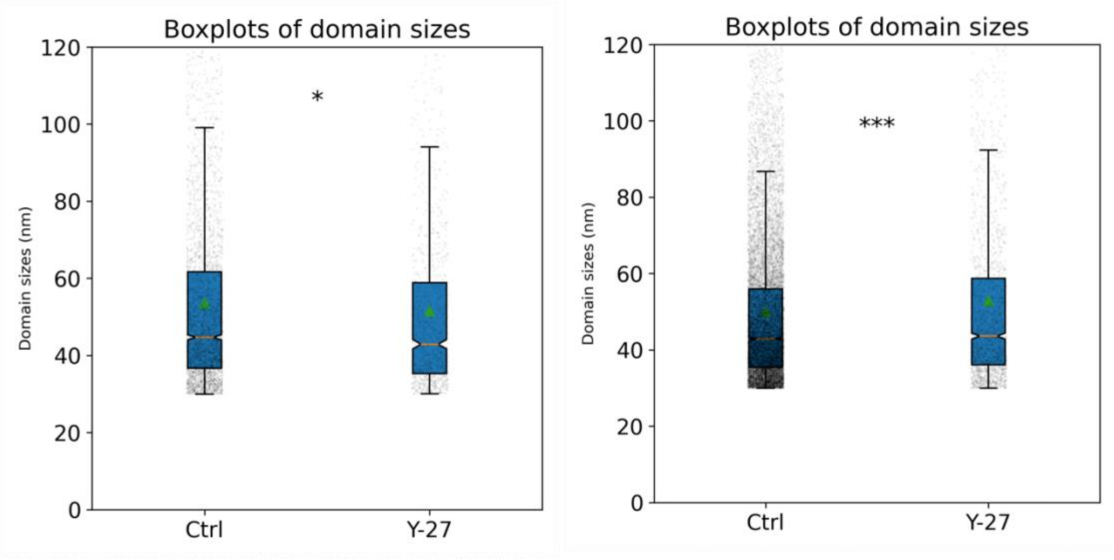

Figure 17: Chromatin domain sizes scale with Y-27 treatment in hMSCs and are affected in tendinosis[1].

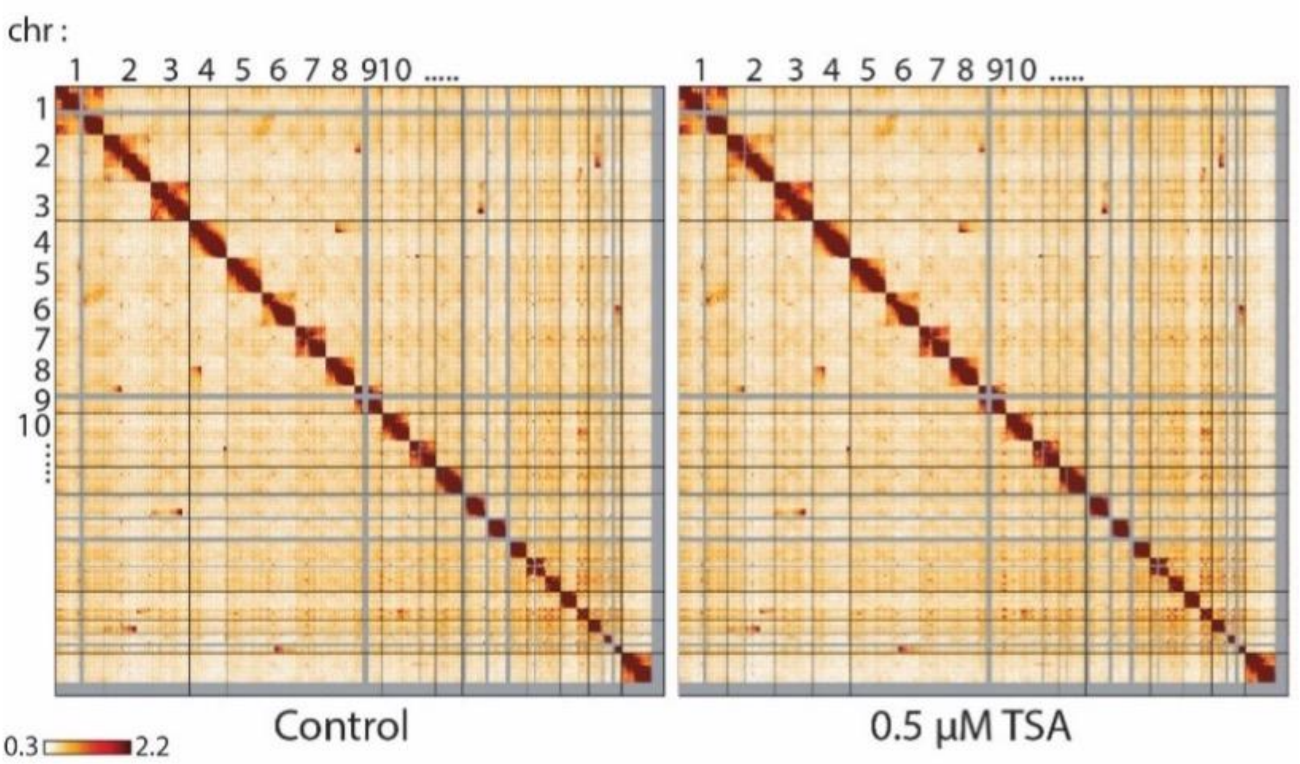

Figure 18: Control and TSA treated Hi-C matrices for all chromosomes.

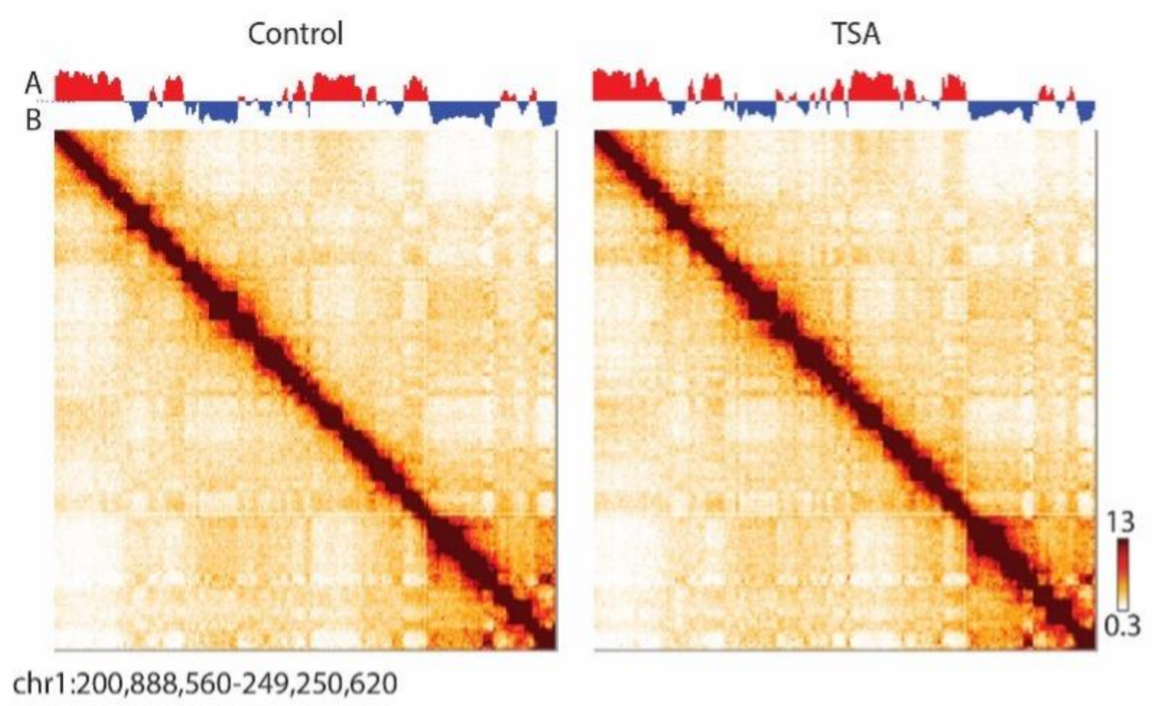

Figure 19: HiC maps do not show any visible changes between control and TSA treated cells for A375 cells.

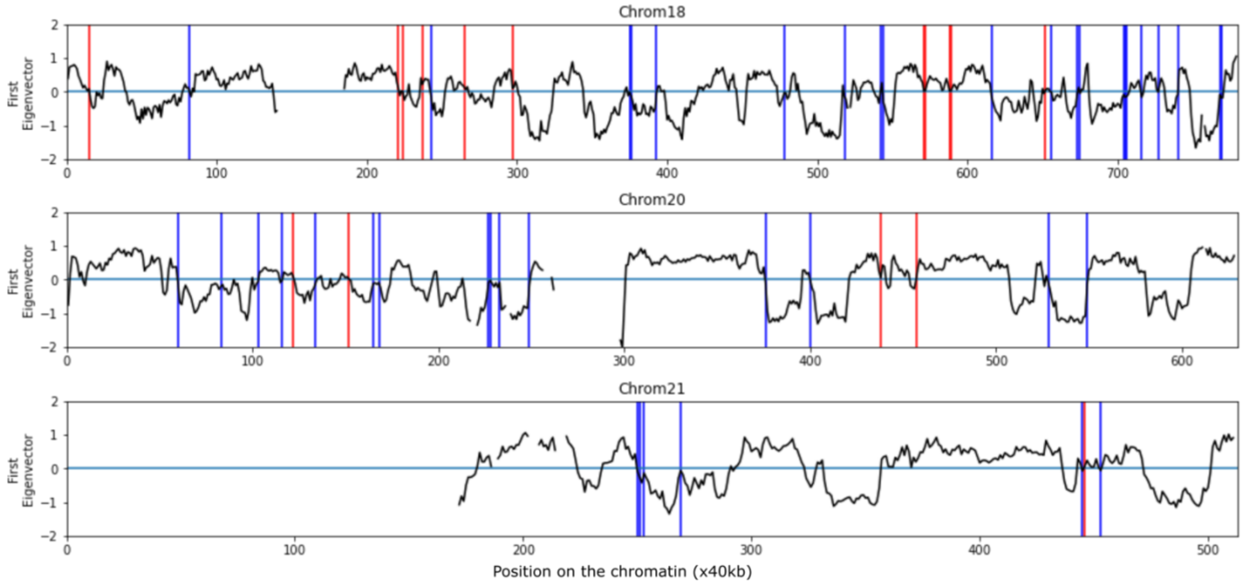

Figure 20: Compartment switches for chr18, 20 and 21 for A375 cells after TSA treatment. The black lines represent the control PC1 with the blue vertical lines representing B to A transition and red vertical lines representing A to B transitions.

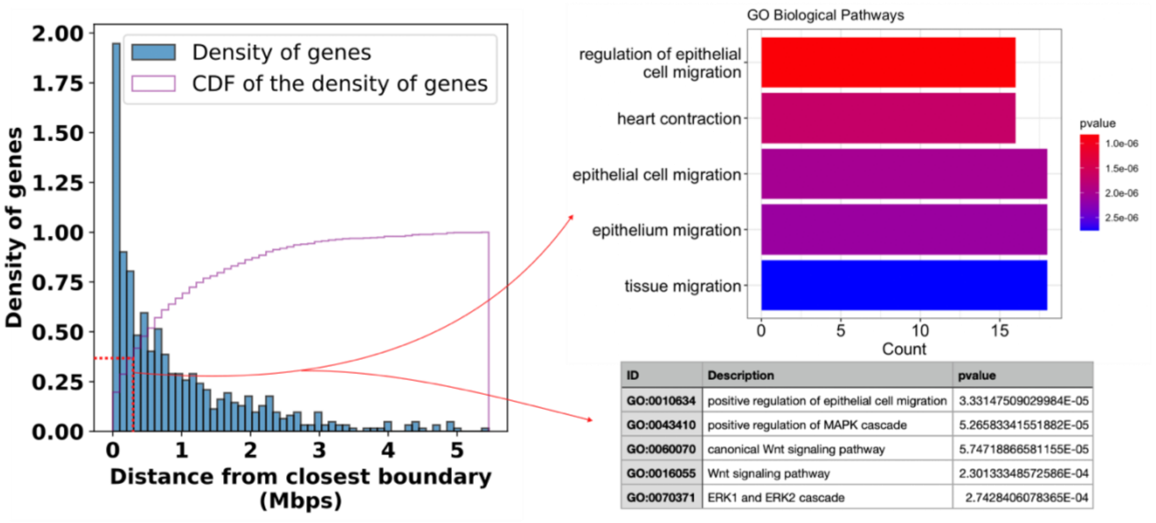

ID	Description	pvalue
GO:0010634	positive regulation of epithelial cell migration	3.33147509029984E-05
GO:0043410	positive regulation of MAPK cascade	5.26583341551882E-05
GO:0060070	canonical Wnt signaling pathway	5.74718866581155E-05
GO:0016055	Wnt signaling pathway	2.30133348572586E-04
GO:0070371	ERK1 and ERK2 cascade	2.7428406078365E-04

Figure 21: Almost 30% differentially regulated genes lie within 300kbps of the domain boundaries. GO analysis of these genes using clusterProfiler shows that migration and ERK, Wnt pathways are still majorly upregulated. Over-Representation Analysis (ORA) used Fisher's exact test (one-sided), with p-values adjusted via the Benjamini-Hochberg (BH) method to control FDR.

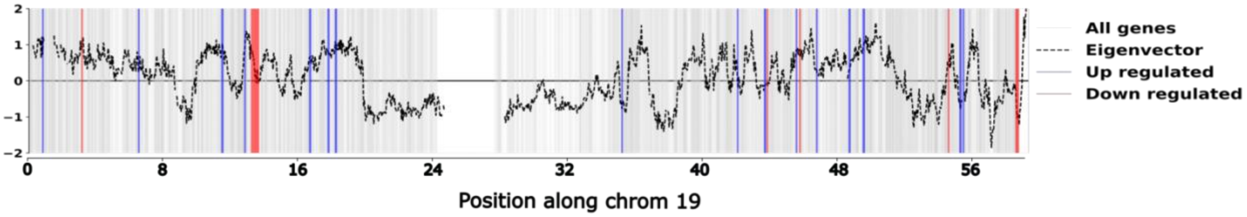

Figure 22: Significantly differentially regulated genes are placed close to the domain boundaries.

Cell line	Source	Chromosomes	Purpose
IMR90	Epigenomics Roadmap ChromHMM	19,20,21	To realize the correct potentials
A375	Experimentally obtained Hi-C contact maps	18,19,20,21	To match with STORM imaging and Hi-C changes upon TSA treatment
hMSC	Epigenomics Roadmap ChromHMM	19,20,21,22	To study the effect of substrate stiffness change and GSK treatment

Table 1: Data for specific cell lines and their usage

Pathway	Genes within 300kb	Ratio of genes found of this pathway found in 300kb /total genes within 300kb	Total number of genes for this pathway/ total number of genes
**Wnt signaling pathway**	FZD4/ TMEM170B/ GSKIP/ STK4/ WNT7B/ CCNYL1/ SOX9/ SCYL2/ EXT1/ CYLD/ LGR4/ CTNND1/ PRICKLE1/ WNT2B	14/227	1.6%
**positive regulation of stress-activated MAPK**	TRAF1/ PER1/ MAPK8IP1/ MAP2K3/ TNIK/ MAP3K3/ TLR9/	7/227	1.5%

Table 2: MAPK and Wnt signaling pathway enrichment within 300kb

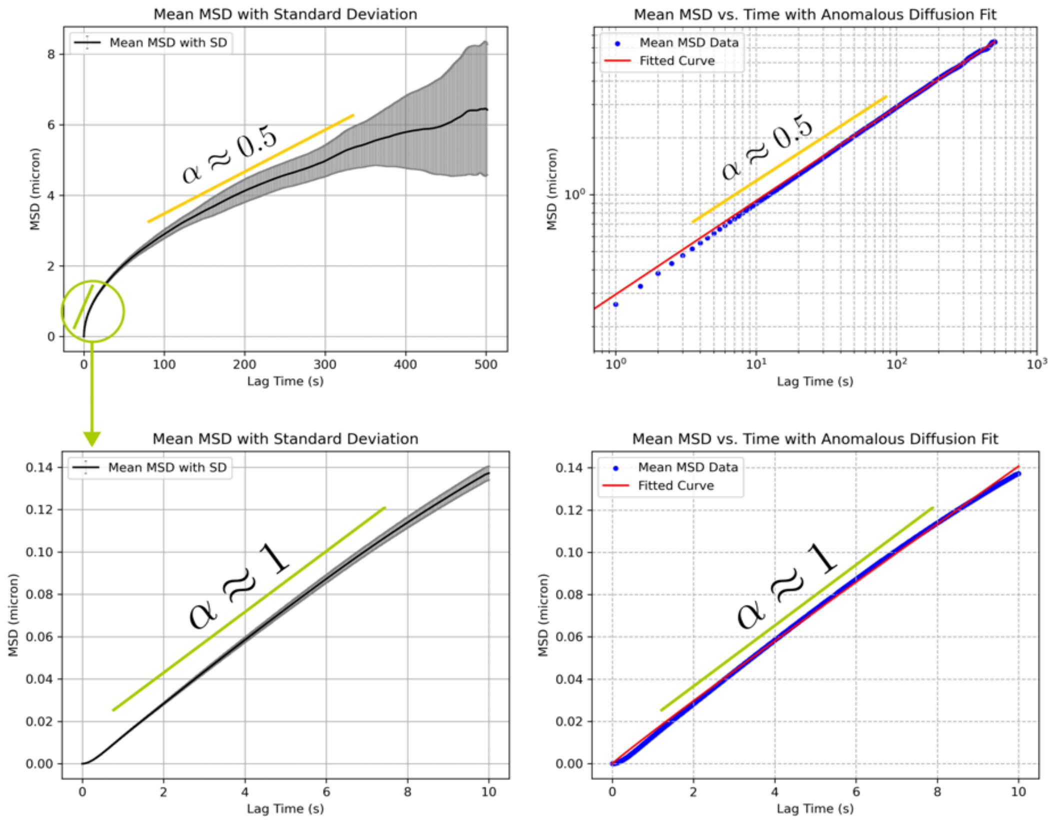

Figure 23: MSD calculations of the polymer showing different diffusive regimes. Top left: MSD calculated over 10 independent runs over the course of ~1hr (real time). Top right: A log-log plot with a linear fit exhibiting diffusion in the $\alpha = 0.49$ regime. The fit produces $D_\alpha = 7 \times 10^{-2}\ \mu m^2/s$. Bottom left: MSD for ~10 seconds. Bottom right: The plot with a linear fit exhibiting diffusion in the $\alpha = 0.98$ regime. The fit produces $D_\alpha = 4 \times 10^{-3}\ \mu m^2/s$.

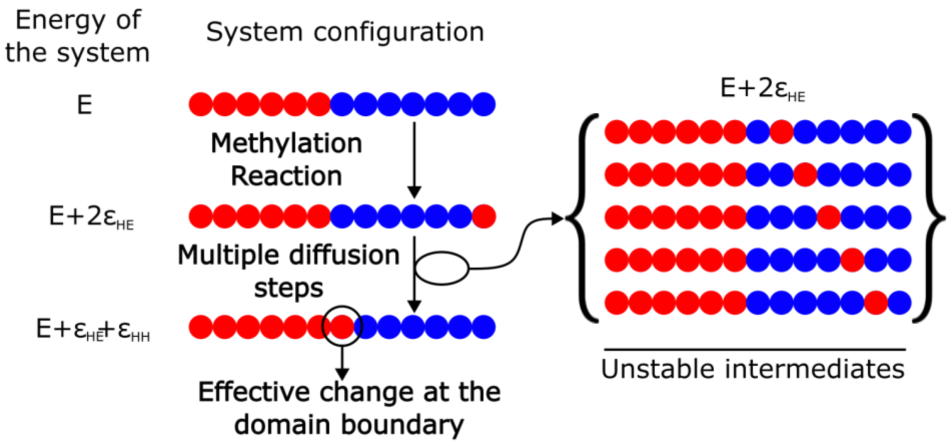

Figure 24: Dynamics favor boundary enrichment of epigenetic changes. Following a methylation event, the altered bead diffuses through the chromatin domain. Its most stable position is at the boundary of the existing domain, as the energy landscape is more favorable due to heterochromatin's affinity for similar chromatin types.

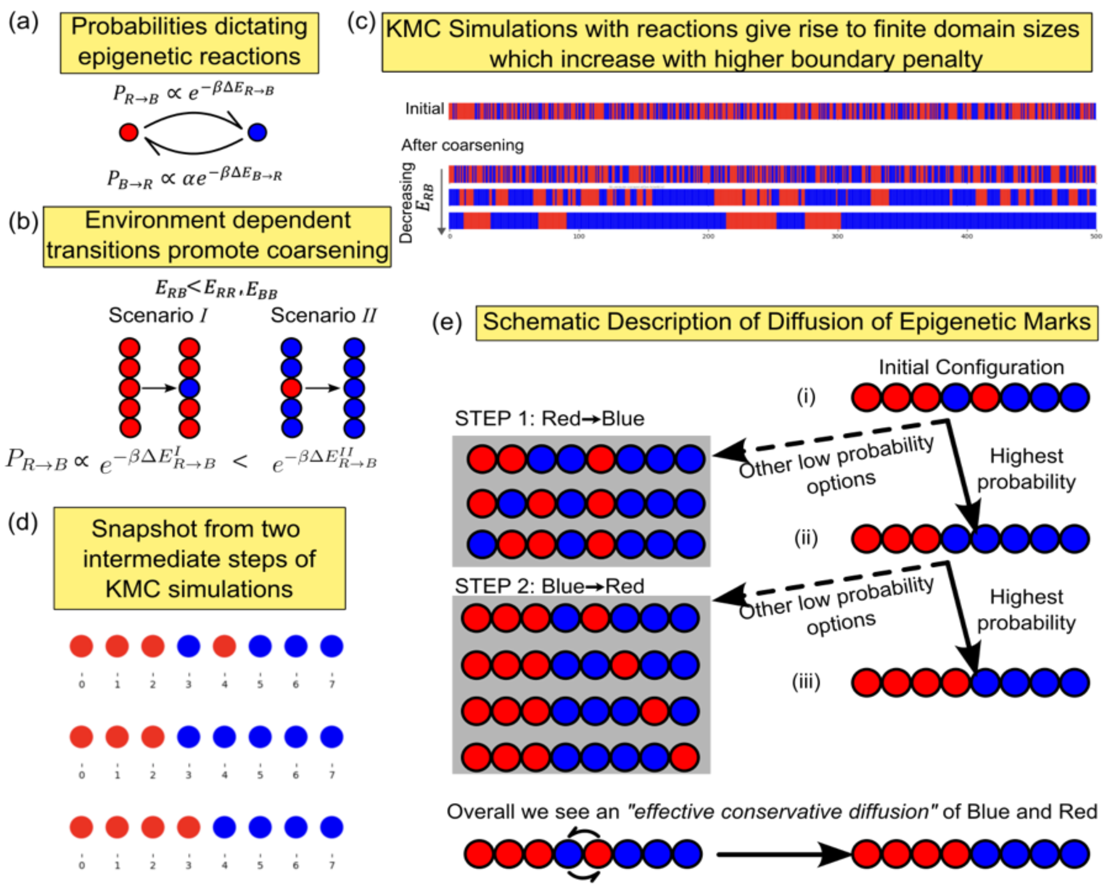

Figure 25: A neighborhood-dependent epigenetic reactions formulation gives rise to diffusion and reactions. a) Epigenetic transition rates which dependent on the epigenetic makeup of the neighborhood and the activity of enzymes. b) Neighborhood dependent reactions lead to coarsening. c)Finite sized domains are formed at steady state. d) Intermediate steps show a resultant conservative exchange of epigenetic marks. e) Effective exchange of epigenetic marks, akin to diffusion of epigenetic marks shown through a two step process.

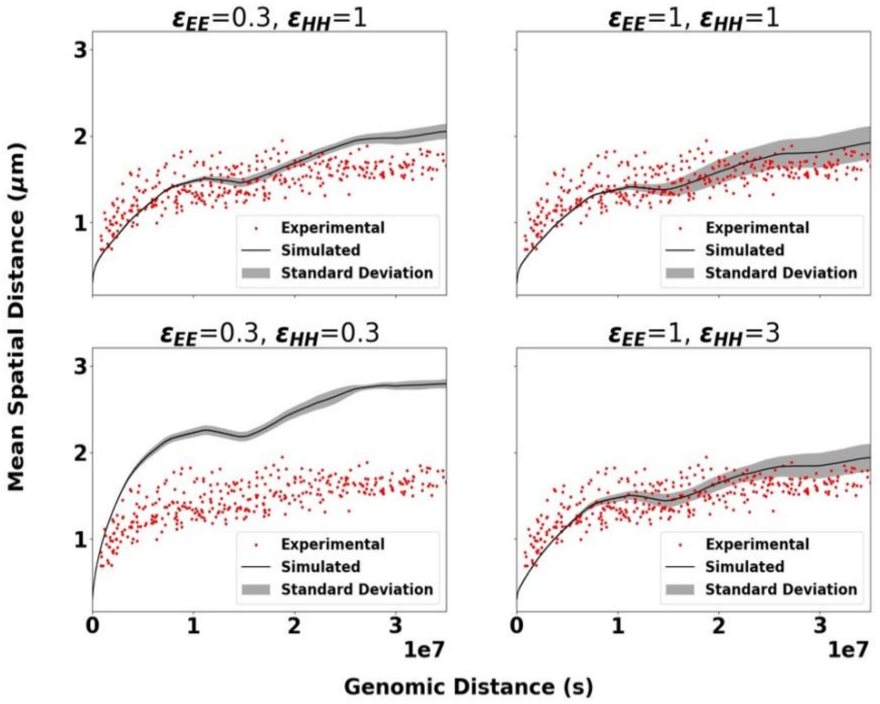

Figure 26: Fitting experimental data with simulations for different attractive potential pairs. The plots show *mean* +/- SD across 10 simulations.

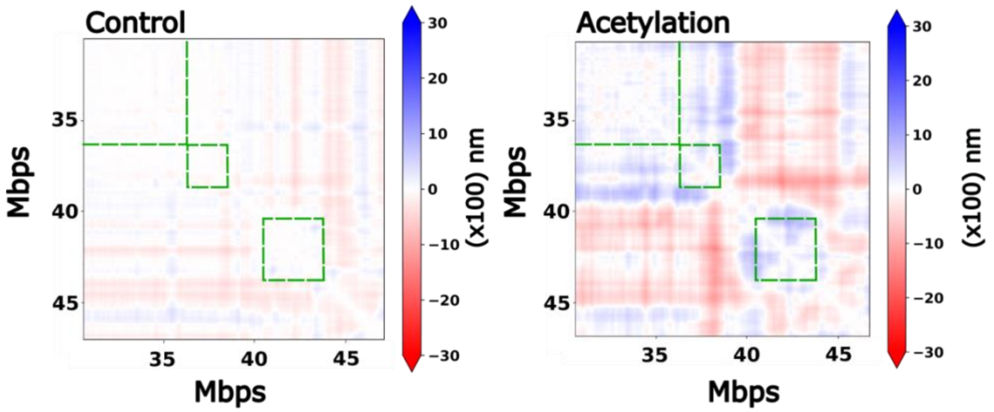

Figure 27: Difference plots for chromosome 19 after 2 hours. The left figure shows control setup, and the right figure shows the acetylated setup. We have subtracted the distance matrix of the final from the initial. The green boxes show the position of the initial domains. The plots show mean map across 10 simulations.
