# Supplementary material for: Polymer model integrates imaging and sequencing to reveal how nanoscale heterochromatin domains influence gene expression
Source: Nat Commun. 2025 Apr 23;16:3816. doi: 10.1038/s41467-025-59001-z (PMC12019571; doi:10.1038/s41467-025-59001-z)
Supplement: Supplementary file 3 — Reporting Summary [file 41467_2025_59001_MOESM3_ESM.pdf]

Corresponding author(s): Vivek B. Shenoy
Last updated by author(s): Mar 19, 2025

Reporting Summary

Nature Portfolio wishes to improve the reproducibility of the work that we publish. This form provides structure for consistency and transparency in reporting. For further information on Nature Portfolio policies, see our Editorial Policies and the Editorial Policy Checklist.

Statistics

For all statistical analyses, confirm that the following items are present in the figure legend, table legend, main text, or Methods section.

n/a	Confirmed	
☐	☒	The exact sample size (*n*) for each experimental group/condition, given as a discrete number and unit of measurement
☐	☒	A statement on whether measurements were taken from distinct samples or whether the same sample was measured repeatedly
☐	☒	The statistical test(s) used AND whether they are one- or two-sided<br>*Only common tests should be described solely by name; describe more complex techniques in the Methods section.*
☒	☐	A description of all covariates tested
☒	☐	A description of any assumptions or corrections, such as tests of normality and adjustment for multiple comparisons
☐	☒	A full description of the statistical parameters including central tendency (e.g. means) or other basic estimates (e.g. regression coefficient) AND variation (e.g. standard deviation) or associated estimates of uncertainty (e.g. confidence intervals)
☐	☒	For null hypothesis testing, the test statistic (e.g. *F*, *t*, *r*) with confidence intervals, effect sizes, degrees of freedom and *P* value noted<br>*Give P values as exact values whenever suitable.*
☒	☐	For Bayesian analysis, information on the choice of priors and Markov chain Monte Carlo settings
☒	☐	For hierarchical and complex designs, identification of the appropriate level for tests and full reporting of outcomes
☒	☐	Estimates of effect sizes (e.g. Cohen's *d*, Pearson's *r*), indicating how they were calculated

Our web collection on statistics for biologists contains articles on many of the points above.

Software and code

Policy information about availability of computer code

Data collection

To perform STORM imaging of the fixed sample, the cells were incubated in blocking buffer containing 10% wt/vol BSA (Sigma) in PBS for 1 h. The samples were then incubated overnight with rabbit anti-H2B (1:100; Proteintech, 15857-1-AP) at 4 °C. Thereafter, the samples were repeatedly washed in PBS, and secondary antibodies (Alexa Fluor 647) were added for imaging. The images were taken on a commercially available ONI (Nanoimager S) STORM microscope system. To ensure optimal photo-switching of Alexa Fluor 647, the imaging buffer followed standard guidelines and consisted of 10 mM cysteamine MEA in Glox solution: 0.5 mg ml-1 1-glucose oxidase, 40 mg ml-1 1-catalase and 10% glucose in PBS. A 640 nm laser was used at a setting of 40% to excite the reported dye (Alex Fluor 647), and a gradual increase in the 405 nm laser was used to reactivate Alexa Fluor 647 in an activator dye to maintain a constant intensity of active fluorophores. An exposure time of 15 ms and 30k frames was used.

Biological replicates of Hi-C were performed on two independent samples of A375 cells, both untreated and treated with 0.5 µM TSA for 2 hr. The first biological replicate was processed with the protocol described previously in Imakaev et. al. at Methods 2019. Briefly, 10 million cells were crosslinked with 1% formaldehyde for 10 min. Crosslinked cells were then suspended in lysis buffer to permeabilize the cell membrane and homogenized. Chromatin was then digested in the nucleus overnight using the DpnII restriction enzyme. The digested ends were filled with biotin-dATP, and the blunt ends of the interacting fragments were ligated together. The DNA was then purified via phenol–chloroform extraction. For library preparation, the NEBNext Ultra II DNA Library prep kit (NEB) was used for libraries with sizes ranging from 200–400 bp. End Prep, Adaptor Ligation, and PCR amplification reactions were carried out on bead-bound DNA libraries. The second biological replicate was processed via the Arima-HiC+ Kit from Arima Genomics following the protocol for Mammalian Cell Lines (A160134 v01), and the libraries were prepared via the Arima recommendations for the NEBNext Ultra II DNA library prep kit (protocol version A16041v01).

Data analysis

STORM images were analyzed using a custom written MATLAB code (made publicly available and citable on GitHub at: https://github.com/ShenoyLab/STORM_Analysis_Parameter_Extraction) to obtain the sizes of the domains.
LAMMPS was used to carry out the molecular dynamics simulations. The analysis was done on python using numpy, pandas and scipy packges.

The codes are provided at GitHub repo: https://github.com/vinayakv161/Epigenetic_diffusion_and_reactions
Softwares used for analysis: MATLAB R2022a (STORM domain analysis), python 3.9
Sequencing was performed on a HiSeq 3000 platform with 150 bp paired-end reads. Sequencing reads were mapped to the human genome (hg19), filtered, binned, and iteratively corrected as previously described[130] via the HiCPro pipeline (https://github.com/nservant/HiC-Pro). Spatial compartmentalization (A/B compartment assignment) was calculated via principal component analysis with 40 kb binned data.

For manuscripts utilizing custom algorithms or software that are central to the research but not yet described in published literature, software must be made available to editors and reviewers. We strongly encourage code deposition in a community repository (e.g. GitHub). See the Nature Portfolio guidelines for submitting code & software for further information.

Data

Policy information about availability of data

All manuscripts must include a data availability statement. This statement should provide the following information, where applicable:

- Accession codes, unique identifiers, or web links for publicly available datasets
- A description of any restrictions on data availability
- For clinical datasets or third party data, please ensure that the statement adheres to our policy

Part of STORM image datasets is originally described in Heo et al.,Nat. Biomed. Eng 2023. A375 STORM images are obtained using ONI (Nanoimage S) using the methodology reported in Heo et al, Nat. BME. 2023. Collection of confocal image datasets is obtained from Leica Sp8 platform with a 63x oil immersion objective. The Hi-C dataset was collected in line with Golloshi et. al., Methods (2018). The RNA-seq was collected as described in Golloshi et. al., EMBO reports (2022).The authors declare that the data supporting the findings of this study are available within the paper and its supplementary information files. The data generated in this study are provided in Source Data file. The sequencing data has been released at GEO accession number: GSE275755.

Research involving human participants, their data, or biological material

Policy information about studies with human participants or human data. See also policy information about sex, gender (identity/presentation), and sexual orientation and race, ethnicity and racism.

Reporting on sex and gender	N/A
Reporting on race, ethnicity, or other socially relevant groupings	N/A
Population characteristics	N/A
Recruitment	N/A
Ethics oversight	N/A

Note that full information on the approval of the study protocol must also be provided in the manuscript.

Field-specific reporting

Please select the one below that is the best fit for your research. If you are not sure, read the appropriate sections before making your selection.

☒ Life sciences ☐ Behavioural & social sciences ☐ Ecological, evolutionary & environmental sciences

For a reference copy of the document with all sections, see nature.com/documents/nr-reporting-summary-flat.pdf

Life sciences study design

All studies must disclose on these points even when the disclosure is negative.

Sample size	STORM image datasets were originally described in Heo et al.,Nat. Biomed. Eng 2023 in which no sample size calculation has been performed before sample collection. 2 Replicates have been done for HiC, RNA-seq experiments, and A375 STORM observations.
Data exclusions	Data corresponding to two nuclei of A375 have been excluded from the analysis. These data points were removed because they significantly deviate, based on a three-standard-deviation limit, from their respective datasets.
Replication	All experimental findings, including chromatin analysis and material characterization, were reliably reproduced.
Randomization	The cells and materials were randomly assigned to the treatment groups or conditions.
Blinding	The investigators were not blinded. All analyses were quantitative in nature, based on either established techniques or on techniques described in the Supplementary Information. There were no subjective or qualitative analyses where decision-making by the researchers would have been required or could have impacted the findings.

Reporting for specific materials, systems and methods

We require information from authors about some types of materials, experimental systems and methods used in many studies. Here, indicate whether each material, system or method listed is relevant to your study. If you are not sure if a list item applies to your research, read the appropriate section before selecting a response.

Materials & experimental systems

n/a	Involved in the study
☐	☒ Antibodies
☐	☒ Eukaryotic cell lines
☒	☐ Palaeontology and archaeology
☒	☐ Animals and other organisms
☒	☐ Clinical data
☒	☐ Dual use research of concern
☒	☐ Plants

Methods

n/a	Involved in the study
☒	☐ ChIP-seq
☒	☐ Flow cytometry
☒	☐ MRI-based neuroimaging

Antibodies

Antibodies used	Rabbit anti-H2B (Proteintech, 15857-1-AP, dilution 1:100), Anti-rabbit Alexa Fluor 647(Abcam, dilution 1:100), DAPI (Thermo Fisher Scientific, D1306, dilution 1:1000), anti-Rabbit H3k9ac (Abcam, ab10812, dilution 1:500).
Validation	All antibodies were validated as described on the manufacturer's website or in our previously published studies.

Eukaryotic cell lines

Policy information about cell lines and Sex and Gender in Research

Cell line source(s)	A375 (CRL-1619) cells were obtained from ATCC
Authentication	NA
Mycoplasma contamination	Cells were verified to be negative for mycoplasma
Commonly misidentified lines (See ICLAC register)	NA

Plants

Seed stocks	NA
Novel plant genotypes	NA
Authentication	NA
